# Illumination discrimination in the absence of a fixed surface-reflectance layout

**DOI:** 10.1167/18.5.11

**Published:** 2018-05-21

**Authors:** Ana Radonjić, Xiaomao Ding, Avery Krieger, Stacey Aston, Anya C. Hurlbert, David H. Brainard

**Affiliations:** radonjic@sas.upenn.edu https://www.sas.upenn.edu/~radonjic/; xiaomao@pennmedicine.upenn.edu; krave@stanford.edu; stacey.j.aston@durham.ac.uk https://staceyaston.com/; anya.hurlbert@ncl.ac.uk https://www.ncl.ac.uk/ion/staff/profile/anyahurlbert.html; brainard@psych.upenn.edu https://color.psych.upenn.edu/people/brainard; Department of Psychology, University of Pennsylvania, Philadelphia, PA, USA; Department of Psychology & Neuroscience Graduate Group, University of Pennsylvania, Philadelphia, PA, USA; Department of Psychology, University of Pennsylvania, Philadelphia, PA, USA; Institute of Neuroscience, Newcastle University, Newcastle upon Tyne, UK; Institute of Neuroscience, Newcastle University, Newcastle upon Tyne, UK; Department of Psychology, University of Pennsylvania, Philadelphia, PA, USA; Neurosciences Graduate Program, Stanford University, Stanford, CA, USA; Department of Psychology, Durham University, Durham, UK

**Keywords:** *color vision*, *illumination perception*, *chromatic illumination discrimination*, *eye fixations*

## Abstract

Previous studies have shown that humans can discriminate spectral changes in illumination and that this sensitivity depends both on the chromatic direction of the illumination change and on the ensemble of surfaces in the scene. These studies, however, always used stimulus scenes with a fixed surface-reflectance layout. Here we compared illumination discrimination for scenes in which the surface reflectance layout remains fixed (fixed-surfaces condition) to those in which surface reflectances were shuffled randomly across scenes, but with the mean scene reflectance held approximately constant (shuffled-surfaces condition). Illumination discrimination thresholds in the fixed-surfaces condition were commensurate with previous reports. Thresholds in the shuffled-surfaces condition, however, were considerably elevated. Nonetheless, performance in the shuffled-surfaces condition exceeded that attainable through random guessing. Analysis of eye fixations revealed that in the fixed-surfaces condition, low illumination discrimination thresholds (across observers) were predicted by low overall fixation spread and high consistency of fixation location and fixated surface reflectances across trial intervals. Performance in the shuffled-surfaces condition was not systematically related to any of the eye-fixation characteristics we examined for that condition, but was correlated with performance in the fixed-surfaces condition.

## Introduction

The visual system receives information about the environment when illumination from the light sources reflects off of objects and reaches the eye. Variations in illumination—both over time and across space—are ubiquitous in natural scenes (Nascimento, Amano, & Foster, 2016; Spitschan, Aguirre, Brainard, & Sweeney, 2016) and can dramatically modulate the light reflected from objects and, consequently, the information available to vision about their physical properties, such as surface reflectance. For this reason, the visual system adjusts its processing of the retinal image across changes in illumination, to maintain relatively stable perception of object colors (Brainard & Radonjić, 2014). Our understanding of the processes that underlie this *color constancy* is mostly based on studies that measure perceived object color across changes in illumination (Hurlbert, 1998; Olkkonen & Ekroll, 2016; Smithson, 2005).

Due to the key role that illumination plays in determining the light reflected from objects to the eye, a complementary approach to studying constancy focuses on illumination perception. Illumination perception has been studied both directly, using tasks in which observers make explicit judgments about the illumination, and indirectly, in paradigms where inferences about the perceptual representation are made on the basis of measurements of other stimulus attributes. Studies involving direct judgments have probed the ability to perceive spatial characteristics of the illumination, including direction (Pont, van Doorn, & Koenderink, 2017; Xia, Pont, & Heynderickx, 2016), diffuseness (Morgenstern, Geisler, & Murray, 2014), and perceived distribution of illumination in space (“the visual light field”, Kartashova, Sekulovski, de Ridder, te Pas, & Pont, 2016; Koenderink, Pont, van Doorn, Kappers, & Todd, 2007; Xia, Pont, & Heynderickx, 2014; see also Schirillo, 2013). In other studies using direct assessments, observers were asked to make explicit asymmetric matches of illumination levels or directions (Khang, Koenderink, & Kappers, 2006; Rutherford & Brainard, 2002; see also Logvinenko & Menshikova, 1994). Studies using indirect judgments mainly focus on developing models of inferred illumination based on measurements of object surface reflectance (Bloj et al., 2004; Boyaci, Doerschner, & Maloney, 2004; Fleming, Dror, & Adelson, 2003; Logvinenko & Maloney, 2006; see Brainard & Maloney, 2011) or object shape (Morgenstern, Murray, & Harris, 2011; van Doorn, Koenderink, Todd, & Wagemans, 2012). Within both approaches, efforts have been made to investigate which image cues support perceptual representations of illumination (Boyaci, Doerschner, & Maloney, 2006; te Pas, Pont, Dalmaijer, & Hooge, 2017). The study of illumination perception is often motivated by its links to object color constancy. However, the topic is also of interest in its own right, as illumination provides important information about environmental conditions, such as time of day or future weather.

Our own work on illumination perception has focused on characterizing human sensitivity to changes in illumination spectrum. Using real illuminated scenes as stimuli, we measured illumination discrimination thresholds across four different chromatic directions of illumination change: yellow and blue, which follow the daylight locus, and red and green, orthogonal to the daylight locus (Pearce, Crichton, Mackiewicz, Finlayson, & Hurlbert, 2014). We found that sensitivity varies across different chromatic directions, with sensitivity in the blue direction being the lowest when stimulus distance is expressed using the CIELUV metric (CIE, 2004).

In a follow-up study (Radonjić et al., 2016), we replicated these results for scenes consisting of real illuminated surfaces as well as for well-matched computer-generated scenes. We found that results obtained for real illuminated scenes generalize well to synthetic scenes, which allows us to take advantage of the parametric control offered by computer graphics to probe aspects of illumination discrimination that would otherwise be difficult to study. For example, using simulated scenes, we showed that the relative sensitivity to different chromatic directions of illumination change depends on the ensemble of surfaces in the scene (Radonjić et al., 2016): when the relative number of yellow and green surfaces in the scene ensemble increased, thresholds for a blue illumination-change direction increased; when the relative number of reddish-blue surfaces in the scene ensemble increased, the thresholds for a red illumination-change direction decreased. Although further research is required to characterize the processes that mediate this change in thresholds, our results indicate that any characterization of sensitivity to illumination changes requires taking into account the ensemble of surfaces in the scene, in addition to the chromatic direction of the illumination changes.

Other recent studies have contributed to better understanding of processes underlying illumination discrimination. For example, Weiss and Gegenfurtner (2016) studied illumination discrimination in conjunction with surface chromatic discrimination, across a wide range of chromatic directions. Using real illuminated scenes, Aston, Radonjić, Brainard, and Hurlbert (2018) measured the variation in illumination discrimination thresholds for different starting locations in illumination chromaticity space. Álvaro, Linhares, Moreira, Lillo, and Nascimento (2017) measured illumination discrimination across blue-yellow directions using images of natural scenes and showed that illumination discrimination thresholds in dichromats are similar to those of normal observers.

**Figure 1:**
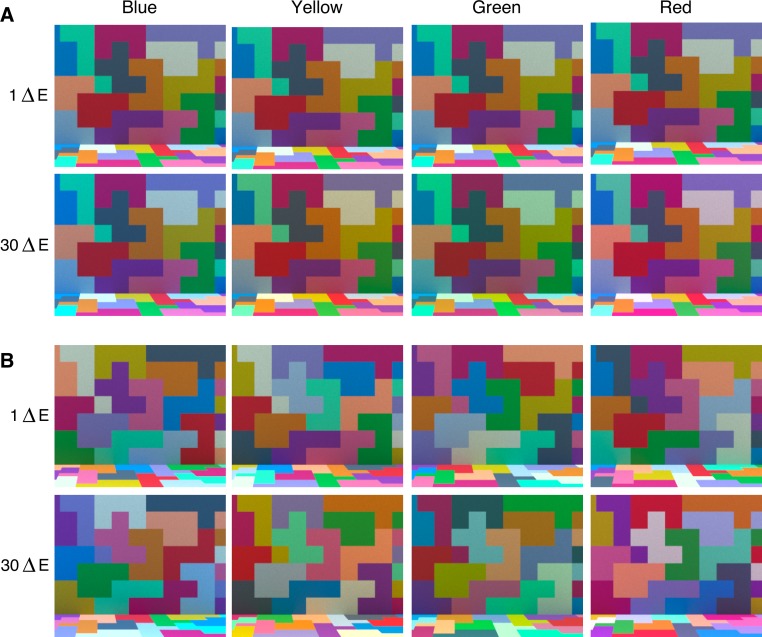
Stimulus scene under different illuminations (Experiment 1). Panel A. Fixed-surfaces condition. Panel B. Shuffled-surfaces condition. For each of the four illumination-change directions we show the stimulus scene rendered under test illuminations, which differ from the target by 1 or 30 (nominal) ΔE units (left eye image from each stereo pair is shown).

In previous studies of illumination discrimination, the surfaces in the scene always remained fixed within each trial (see Figure 1A), so that the experiments probed the ability to discriminate temporal illumination changes within an otherwise fixed environment. The more general situation is one in which both the illumination and the surfaces in the scene change, as occurs when one turns around outside or steps from one room into another. Here we report experiments that extend our study of illumination discrimination to a case where the spatial distribution of surface reflectances within a scene varies concurrently with illumination changes (see Figure 1B). We take advantage of our ability to generate rendered images of synthetic scenes, which enables us to independently manipulate in software both the spectral power distribution of the illumination and the spectral reflectance functions of the surfaces in the scenes.

The work we report here provides further insight into processes that underlie illumination discrimination. For example, it is currently unclear how the visual system differentiates between illuminations. One possibility is that to detect changes in illumination, observers track changes in the light reflected from a subset of surfaces in the scene. In this case, performance would be significantly impaired when tracking individual surfaces becomes more difficult, as when the surface layout varies across scenes. Alternatively, observers may form a global estimate of scene illumination based on the spatial average of the light reflected to the eye. Judgments based on such an estimate would be robust to variations in surface reflectance layout that do not affect the spatial average of the surface reflectances.

## Experiment 1

To establish whether and to what extent sensitivity to changes in illumination is affected when the surfaces in the scene vary as the illumination changes, we compared observers' performance on the illumination discrimination task in a *fixed-surfaces* condition to that in the *shuffled-surfaces* condition. In both conditions, the sensitivity was measured for four different chromatic directions of the illumination change (blue, yellow, red, and green) and the geometric structure of the stimulus scene was the same. The stimulus scenes were rendered images depicting a room whose wall and floor were covered with rectangular surfaces, which varied in reflectance. In the fixed-surfaces condition, the reflectance assigned to each visible surface in the room remained unchanged across all stimulus scenes, and only the illumination varied. In the shuffled-surfaces condition, the surface layout remained the same, but the reflectances assigned to surfaces were shuffled randomly as the illumination was varied. Although across scenes the reflectance of individual surfaces varied, the mean scene reflectance was kept roughly constant. This was achieved by assigning approximately the same surface area in each scene to each specific reflectance.

Visual examination of our stimuli (Figure 1) suggests that detecting changes in illumination across successive scenes in which the surfaces shuffle is considerably more challenging than when the surfaces are fixed: Figure 1 shows examples of stimulus scenes for the two conditions. In each panel, pairs of test illuminations that differ by 29 CIELUV ΔE units are shown across the four chromatic directions of illumination change (1 vs. 30 ΔE units, relative to target illumination). The difference between the test illuminations is much easier to see in the fixed-surfaces condition (panel A) than in the shuffled-surfaces condition (panel B). This is consistent with the results we present below: Illumination discrimination thresholds were higher in the shuffled-surfaces condition than in the fixed-surfaces condition.

### Methods

General methods for Experiment 1 were similar to those from our previous study (Radonjić et al., 2016). Therefore, some sections of the Methods here are taken verbatim from our previous publication.

#### Preregistration

Before the start of the data collection, we registered a document that described the experimental design and the data analysis plan for this study. This document was time-stamped and frozen at the time of submission. It is publically available on the Open Science Framework: https://osf.io/s65ef/ (see also https://osf.io/csuza/). Departures from the preregistered plan are summarized in the Appendix.

#### Apparatus

Stimuli were presented stereoscopically on a custom stereo-display apparatus in an otherwise dark room. The apparatus consisted of a pair of calibrated LCD color monitors (24-in. NEC MultiSync PA241W; NEC Display Solutions, Itasca, IL). The monitors were driven at a pixel resolution of 1,920 × 1,200, a refresh rate of 60 Hz, and with 8-bit resolution for each RGB channel via a dual-port video card (NVIDIA GeForce GT120; NVIDIA, Santa Clara, CA). Observers viewed the displays through two rectangular apertures (2.7 × 2.5 cm) in a single black metal plate. The position of the apertures relative to the screens was such that the left screen was visible only to the left eye while the right screen was visible only to the right eye. The optical distance of each monitor to the eye was 76.4 cm. Additional detail about the apparatus is available elsewhere (Lee & Brainard, 2014). The host computer was an Apple Macintosh with an Intel Xeon Quad-Core processor. The experimental programs were written in MATLAB (MathWorks, Natick, MA), using routines from the Psychophysics Toolbox (Brainard, 1997; Pelli, 1997, http://psychtoolbox.org) and mgl (http://justingardner.net/doku.php/mgl/overview).

#### Stimulus scenes

The stimuli were computer-graphics simulations of a scene depicting a room viewed through a window-like opening in the front wall. The room ceiling was white, the back and side interior walls and the floor were light gray (reflectances of Macbeth color checker chart samples: row 4, columns 1 and 2, respectively; http://www.babelcolor.com). The exterior surface of the room was black (reflectance 0 at all wavelengths). The room was illuminated with an area light, which covered the entire surface of the ceiling and created relatively diffuse illumination across the simulated room. The spectrum of the area light varied across the stimulus set (as described below).

The portions of the back wall and the floor that were visible through the opening of the room were covered with square colored tiles (Figure 1). There were approximately 77 tiles covering the wall (7 × 10) and 63 tiles covering the floor (7 × 9; the numbers are summed across right and left stereo-images). Each tile was assigned a reflectance value from a set of 14 reflectance samples (a subset of those used in our previous study, Radonjić et al., 2016). We aimed to assign each reflectance in the set to approximately the same number of tiles (∼5.5 tiles per reflectance for the wall, ∼4.5 tiles per reflectance for the floor; for most tiles at the edges of a scene only a portion of the tile's surface was visible). The tiles that were assigned the same reflectance were often placed next to one another and grouped to create an irregular geometric pattern of constant surface reflectance.

Across conditions we varied the surface reflectance layout by varying which reflectance sample was assigned to which tiles, while keeping the geometry of the scene fixed. In the fixed-surfaces condition, the reflectance-to-tile assignment remained the same across all stimulus scenes. In the shuffled-surfaces condition the assignment was shuffled randomly for each rendered scene, while preserving grouping of tiles by constant surface reflectance (see Figure 1). Although in the shuffled-surfaces condition the reflectance layout changed across scenes, the relative area mapped onto a single reflectance in the scene remained relatively constant (as, across scenes, each surface reflectance was assigned to approximately the same number of tiles on the wall and floor). We were, therefore, able to maintain approximately the same mean scene reflectance as we shuffled the reflectances of individual tiles.

Our stimulus scenes did not contain any discrete objects. In our previous work, we showed that sensitivity to changes in illumination does not change significantly when objects—either novel (e.g., geometric shapes) or familiar (e.g., fruit)—are introduced in the experimental scene (Pearce et al., 2014).

#### Experimental illuminations

We used a set of 201 illumination spectra in the experiment: one target illumination and 200 test illuminations (50 in each of the 4 illumination-change directions). The illumination spectra were close to equiluminant but varied in chromaticity. They were identical to the spectra used in our previous study (Radonjić et al., 2016), where we also describe in detail how this set of experimental illuminations was constructed. The target illumination was a metamer of daylight illumination of correlated color temperature of 6700 K (D67). The test illuminations varied along four chromatic directions relative to the target: blue and yellow, which followed the daylight locus, and red and green, which were orthogonal to the daylight locus. The chromaticities of the test illuminations were chosen so that the difference between the test and the target illumination increased gradually in steps of approximately 1 CIELUV ΔE unit. Thus, along each chromatic direction, the nominal difference between the target and the test illumination ranged from 1 to 50 ΔE. The actual difference between each illumination and the target differed slightly from this nominal value. For each test illumination, we computed the actual distance from the target in ΔE (by converting the illumination spectra into CIELUV values via CIEXYZ (CIE, 1932) and using the target illumination *XYZ* value as the white point for the conversion). A table specifying the exact distances is available in the online supplement. In the data analysis, we use actual, rather than nominal ΔE values. Note that these ΔE values, as well as all others presented in this paper, are computed based on values for all three CIELUV dimensions and thus incorporate both chromatic and luminance differences.

#### Stimulus set

Each stimulus scene was rendered from two different viewpoints, corresponding to the left and right eyes, by shifting the camera position used to render by ±3.2 cm. We will use the term *scene* to refer to the three-dimensional description of the stimulus and the term *image* to refer to two-dimensional rendered images displayed on the monitors. For each scene, there were two rendered images, one for the left eye and one for the right eye.

For each condition (fixed- and shuffled-surfaces), we rendered 230 scenes: 30 target scenes, which were illuminated by the target illumination (30 scenes × 1 target illumination) and 200 test scenes, each illuminated by a different test illumination (1 scene × 50 test illuminations × 4 chromatic directions of illumination change).

In the shuffled-surfaces condition we rendered a pool of 30 different versions of the target scene (each with a different surface reflectance assignment) to avoid repetition of the same assignment across trial intervals and minimize repetition across trials. On each trial, two different target scenes were drawn randomly from this pool (one for the target interval and one for one of the two comparison intervals). To maintain parallel design, we rendered 30 target scenes in the fixed-surfaces condition as well and presented two different scenes, randomly drawn from the pool, on each trial. In the fixed-surfaces condition all target images had identical reflectance-to-tile assignment and differed only due to small run-to-run variations in the stochastic ray-tracing performed by the rendering algorithm. In the shuffled-surfaces condition, each of the 30 target scenes (and each of the 200 test scenes) was rendered using a different reflectance-to-tile assignment.

#### Stimulus generation

The scenes were modeled in Blender (open-source software for 3D modeling and animation, https://www.blender.org/manual/) and rendered in Mitsuba (open-source software for physically-based rendering, https://www.mitsuba-renderer.org/), using a path-tracer integrator (which models interreflections between surfaces) and a low discrepancy sampler (sample count: 320). Rendering was managed using RenderToolbox3 (Heasly, Cottaris, Lichtman, Xiao, & Brainard, 2014, https://github.com/DavidBrainard/RenderToolbox3/wiki), which enabled us to specify the spectral reflectance of each surface and the spectral power distribution of the illumination for each scene.

Each image of the stereo-pair was initially rendered as a 31-plane hyperspectral image. This was then converted into a three-plane LMS image by computing the pixel-by-pixel excitations that would be produced in the human L-, M-, and S-cones, using the Stockman–Sharpe 2° cone fundamentals (CIE, 2007; Stockman & Sharpe, 2000). We used standard monitor calibration and correction methods to convert each LMS image into an RGB image for presentation (Brainard, Pelli, & Robson, 2002). Monitor calibration measurements included the spectral power distributions of the monitor's R, G, and B primaries as well as gamma function of each monitor channel. Calibration measurements were made using a PR-670 SpectraScan radiometer (Photo Research Inc, Syracuse, NY). All of the rendered images were scaled by a common constant to maximize the fraction of the display gamut used by the stimulus set. The effect of this scaling is equivalent to increasing the illumination irradiance by a common factor across all of the scenes and preserves the relative equivalence of illumination irradiance across scenes. The proximal luminance of the displayed images is described below.

#### Task

**Figure 2:**
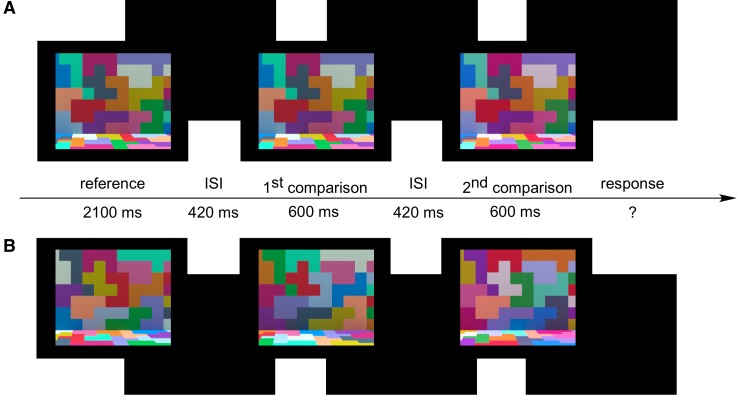
Trial sequence example (Experiment 1). Panel A. Fixed-surfaces condition. Panel B. Shuffled-surfaces condition. On each trial the observer viewed the scene rendered under target illumination (reference interval), followed by two comparison scenes, each rendered under different illumination (comparison intervals). Each stimulus interval was separated by a dark interstimulus interval (ISI). Labels in the diagram indicate the duration of each interval in milliseconds (these were identical across conditions). In both panels the scene shown in the first comparison interval is rendered under the target illumination, while the scene shown in the second comparison interval is rendered under red test illumination (30 ΔE). In the experiment, the order of scenes presented in the two comparison intervals was randomized on each trial. Figure shows left image of the stereo-pair only. Images are tone-mapped for illustration purposes (as described in Radonjić et al., 2016).

On each trial of the experiment the observers saw a sequence consisting of three stimulus intervals, separated by a 420-ms-long dark interval (Figure 2). First the scene rendered under the target illumination was presented for 2,100 ms (the reference interval). Then, two comparison scenes, rendered under different illuminations, were presented for 600 ms each (the comparison intervals). On each trial, one of the comparison scenes was rendered under the target illumination, while the other one was rendered under one of the test illuminations. After the second comparison interval, the screens turned dark and the observer responded whether the illumination for the first or the second comparison scene was most similar to the target scene illumination.

The order of scene presentation in the comparison intervals (target illumination scene first versus target illumination scene second) was randomized on each trial. The degree of change between the target and the test illumination on each trial was determined via a staircase procedure (described below). Observers responded using a game controller and could take as much time as they needed to respond.

On each trial two different target images were drawn randomly from a set of 30 target images: one of these was presented in the target interval while the other was presented in one of the comparison intervals. In the fixed-surfaces condition these two target images were essentially identical (up to small differences due to the rendering noise described above). In the shuffled-surfaces condition the two target images had a different surface-reflectance layout (see Figure 2). Thus, in the shuffled-surfaces condition a different surface reflectance layout was used in all three trial intervals (target and two comparison intervals). In the fixed-surfaces condition, the same surface reflectance layout was used in all trial intervals (as well as across all trials).

#### Proximal stimulus

The size of each image was 18.7° × 15.7° of visual angle (25.1 × 21 cm). In the fixed-surfaces condition the target image mean luminance was 17 cd/m^2^ and *xy* chromaticity was (0.326, 0.327). These values were obtained by averaging all pixels in the left and the right image for one target scene, and then taking the mean across all 30 target scenes. Variations in chromaticity and luminance across different target images were negligible in the fixed-surfaces condition (*SD* < 0.003 for luminance; < 0.0001 *x* and *y*). In the shuffled-surfaces condition, the chromaticity and luminance varied slightly across the target images, due to the variation in reflectance layout. Across the 30 target images for the shuffled-surfaces condition, the mean luminance was 17 cd/m^2^ (*SD*: 0.49) and the mean *xy* chromaticity was (0.324, 0.326), *SD*s (0.004, 0.004).

The reader may wonder why there was any variation in the chromaticity and luminance of the target images in the shuffled-surfaces condition. In a flat, two-dimensional image in which all surfaces receive the same illumination and each surface reflectance sample covers precisely the same scene area, shuffling the reflectance-to-surface assignment under a fixed illumination would not cause changes in average image luminance and chromaticity. However, our scenes were three-dimensional, the area each reflectance covered across the scenes was equated approximately, and the illumination, which came from an area light on the ceiling, was not perfectly uniform across the scene (e.g., surfaces on the floor received more illumination than surfaces on the back wall; see Figure 1). Due to these factors, rendering different scene reflectance layouts under a fixed illumination yields stimulus images that differ slightly in mean chromaticity and luminance, as noted above.

To quantify image variation due to shuffling, we first computed the mean *XYZ* value for each of the 30 versions of the target image. We converted these mean *XYZ* values to CIELUV, using an image-based target illumination *XYZ* value as the white point for conversion.^1^ We then computed the pairwise ΔE differences across all 30 versions of the target image. The mean difference computed across images was 2.69 ΔE (*SD*: 1.32; maximum: 7.16). For comparison, the mean difference across target images in the fixed-surfaces condition was smaller than 0.01 ΔE (maximum smaller than 0.02). For each condition, we also computed the relative distance (in CIELUV ΔE) of each test image from the mean target image, averaged across all 30 target images. Tables specifying these distances are available in the online supplement for each experiment. These tables differ from the lookup table used to compute thresholds (as noted above, those tables were computed from illumination spectra using target illumination *XYZ* as the white point in *XYZ* to LUV conversion).

#### Experimental procedures

At the beginning of the first experimental session, observers were read comprehensive experimental instructions (these are provided verbatim in the online supplement). At the beginning of each subsequent session, they received abbreviated instructions (taken verbatim from the full instructions) reminding them of their task in the experiment, how to provide responses via the game controller, and how to take breaks during the course of the experiment.

Each observer completed four experimental sessions, each run on a different day. During a session, observers completed one block of either the fixed-surfaces condition (first and third session) or the shuffled-surfaces condition (second and fourth session).

Each block consisted of 12 interleaved 1-up-2-down independent staircases: three for each of the four directions of illumination change. Each staircase had a different starting point. For one staircase the starting point was chosen randomly from the 11–20 ΔE units interval, for one it was chosen randomly from 21–30 ΔE units interval, and for the last it was chosen randomly from 31–40 ΔE units interval. The staircase step size was set to 15 nominal ΔE units at the beginning of each trial and changed after the first four reversals (to 10 ΔE after the first, 5 ΔE after the second, 3 ΔE after the third, and 1 ΔE after the fourth reversal). Each staircase terminated either after the eighth reversal or after 50 trials, if the eighth reversal had not been reached. Within a block, the staircases were presented in an interleaved manner, with the staircase used on each trial chosen at random from the set of staircases that were not yet finished.

At the start of each session, observers completed 12 training trials per illumination direction (three initial trials from a staircase initiated with a test illumination drawn from a 31–40 ΔE interval and with the reversal step size fixed at 10 ΔE steps). The training trials (fixed or shuffled) corresponded to the condition being run in the session. Observers were aware that the first 12 trials were training trials, and these trials were not analyzed.

Each session typically lasted about an hour. To prevent fatigue, the experiment was paused approximately midway through each session (after 20–25 min), and observers took 5–10 min break before continuing.

At the end of the experiment the observers completed a brief questionnaire in which they were asked to describe in their own words how they approached the experimental task, whether they noticed any differences across sessions, and also to provide any additional impressions and/or comments they might have about the experiment. Scanned questionnaires with observers' responses are available in the online supplement.

Due to technical difficulties (stimulus image was likely to have been shown on only one display while the other display was black), the first fixed-surfaces session had to be repeated for one observer (“10400”). The data from the session in which the stimulus was not correctly displayed was discarded.

#### Exclusion criteria

It is possible for an observer's sequence of responses to lead to an estimated threshold larger than 50 ΔE (nominal) units; that is a threshold estimate outside of the stimulus range. For the fixed-surfaces condition, in which thresholds typically fall between 5 and 15 ΔE units, such a result would be indicative of an observer who responded randomly. We therefore preregistered a plan to exclude from the analysis any observer whose threshold estimate for any chromatic direction in either fixed-surface session was larger than 50 nominal ΔE units, and to recruit additional observers to replace those excluded. One observer (see Observers) was excluded and replaced for this reason. We also preregistered a plan to exclude and replace any observers whose thresholds fell below 1 ΔE unit for any condition (fixed or shuffled) for any direction. No observers were excluded for this reason.

Further, we preregistered a plan to exclude from analysis, but not replace, observers whose thresholds for any illumination-change direction in the shuffled-surfaces condition were outside the stimulus range for both experimental sessions. The data of one observer (see Observers) was excluded from the analysis for this reason.

#### Observers

Eleven observers participated in Experiment 1 (five females and six males; age: 22–37). They all received either course credit or $10/hour compensation for their participation. All observers had normal or corrected-to-normal visual acuity (both eyes 20/40 or better, as assessed via Snellen chart), normal color vision (100% correct score on Ishihara color plates; Ishihara, 1977), and normal stereo-vision (depth-discrimination thresholds of 2 cm as assessed via a custom lab procedure; Lee & Brainard, 2014). One observer (male, age 37) was excluded from the experiment after his third session because his thresholds in the fixed-surfaces condition fell outside of the stimulus range (for both green and yellow chromatic direction, in the second fixed-surfaces session). For one observer (female, age 27) thresholds in two illumination-change directions (yellow and red) in the shuffled-surfaces condition fell outside of stimulus range in both sessions. We did not replace this observer with a new one; rather, we take this as an indicator that in the shuffled-surfaces condition, the illumination discrimination task is too difficult for some observers.

All experimental procedures were approved by the University of Pennsylvania's Institutional Review Board and were in accordance with the World Medical Association Declaration of Helsinki.

#### Online supplement

For all experiments we report, the online supplement (http://color.psych.upenn.edu/supplements/illuminationdiscriminationshuffled/) provides tables specifying the difference between the target and test illuminations in CIELUV ΔE, stimulus description (a Blender file specifying stimulus geometry, surface reflectance functions, illumination spectra and RenderToolbox3 files used for rendering for in each experiment), instructions verbatim for each experiment, and the individual observer data.

#### Data analysis method

We extracted illumination discrimination thresholds from the staircase data following the same methods as in our previous study (Radonjić et al., 2016). For each chromatic direction of the illumination change, we first aggregated the trials across all three staircases, ordered them by illumination-change step size (ΔE), and then grouped them into bins (10 trials per bin, with the last bin containing all the remaining trials). For each bin, we computed the mean illumination-change value and the corresponding mean percent correct (over the binned trials). We then fitted a psychometric function (cumulative Weibull) to this data and extracted a threshold by finding the illumination-change value that corresponded to 70.71% correct identification (a recommended threshold value for the 1- up–2-down staircase procedure; Wetherill & Levitt, 1965). The Weibull functions were fit using the maximum likelihood methods provided in Palamedes Toolbox (Version 1.8.0, Prins & Kingdom, 2009, http://www.palamedestoolbox.org/). In the fitting procedure, the guess rate parameter was fixed at 0.5 (chance-level performance for our task) and the lapse rate parameter was allowed to vary between 0 and 0.05.

In each condition, the final threshold value for each illumination direction and observer was obtained by averaging thresholds across the two sessions. In the shuffled-surfaces condition, thresholds for one or more illumination-change directions fell outside of the stimulus range in one but not both sessions for three out of nine observers. Rather than excluding these observers from the analysis, we followed our preregistered procedure of setting their out-of-range threshold to be equal to the maximal test illumination value for the corresponding illumination-change direction. This value represents a lower bound on observers' performance in the session, and we use it as an approximation of the true threshold value.

Further, we analyzed observers' performance in the experiment via a three-way repeated measures analysis of variance (ANOVA) with condition (fixed vs. shuffled surfaces) and illumination-change direction (blue, green, red, and yellow) as fixed within-subject factors and observer as a random factor.

### Results

We previously established that when the surfaces in the scene remain fixed, illumination discrimination thresholds fall well within the range of illumination variations we used (Radonjić et al., 2016). There was no guarantee, however, that this would be the case for the shuffled-surfaces condition. We found that for the majority of our observers (nine out of 10) the thresholds in each illumination-change direction in this condition were within the stimulus range in at least one session. For three out of the remaining nine observers the thresholds in some illumination-change directions fell outside of the stimulus range in one session (blue for one observer; blue, green, and red for another observer; all four directions for the remaining observer). The procedure for estimating thresholds when a threshold fell out of range in one of the two sessions is described in Methods.

**Figure 3:**
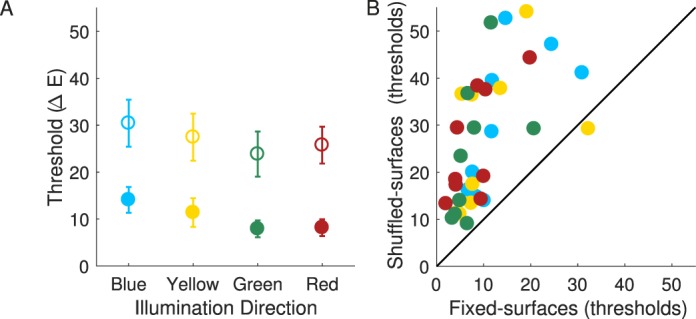
Experiment 1 Results. Panel A. Mean illumination discrimination thresholds (in ΔE, averaged over observers) for the fixed-surfaces (filled circles) and shuffled-surfaces condition (open circles) are shown for four chromatic directions of illumination change. Error-bars indicate ± 1 SEM (where error-bars are not visible, they are smaller than the plotted points). Panel B. For each observer and illumination-change direction, thresholds in the shuffled-surfaces condition are plotted against thresholds in the fixed-surfaces condition (symbol colors correspond to illumination-change direction).

Figure 3A compares observers' thresholds in the fixed- and shuffled-surfaces conditions for the nine observers whose data were retained in the analysis. For each chromatic direction of the illumination change, the filled circles show the mean thresholds, averaged across observers, for the fixed-surfaces condition, while the open circles show mean thresholds for the shuffled-surfaces condition. Clearly, shuffling the surfaces greatly increased the difficulty of the illumination-discrimination task (main effect of condition: *F*(1, 8) = 21.12, *p* = 0.002. Overall thresholds increased by 16.5 ΔE units (26.9 ΔE in the shuffled- vs. 10.4 ΔE units in the fixed-surfaces condition). Note that this increase in thresholds cannot be accounted by the variation in stimulus image chromaticity due to shuffling (which we estimate to be ∼2.7 ΔE on average, based on the variation across the set of the target images; see Methods).

The increase in thresholds from fixed- to shuffled-surfaces condition held for all but one illumination-change direction for one observer. This is illustrated in Figure 3B, which shows individual observer thresholds for each direction in the shuffled-surfaces condition against their corresponding values in the fixed-surfaces condition: All but one data point lie above the diagonal (identity line), indicating the pervasive nature of the increase. The degree to which surface shuffling affected performance did vary across observers: from 4.3 to 30.5 ΔE, Observer × Condition interaction, *F*(8, 71) = 16.17, *p* < 0.001; neither the main effect of observer nor other Observer interactions were significant.

Although thresholds are considerably higher in the shuffled-surfaces condition, our observers performed at above-chance levels. We established this by simulating the performance of 5,000 observers who complete the task by responding randomly (two simulated sessions per simulated observer). We then extracted thresholds for each simulated session and aggregated these across sessions following the same exclusion criteria and data analysis procedures as in the main experiment (see Methods). For 4,998 out of the 5,000 simulated observers, thresholds fell out of stimulus range in both sessions in at least one illumination-change direction (as compared to 1 out of 10 for our actual observers). In other words, 99.96% of randomly responding observers would have been excluded from the analysis based on our exclusion criteria and analysis method.

Consistent with our previous findings (Pearce et al., 2014; Radonjić et al., 2016), thresholds differed significantly across different chromatic directions of illumination change: main effect of direction: *F*(3, 24) = 6.27, *p* = 0.003. The pattern of threshold variations across illumination-change directions across the two conditions was essentially identical (Figure 3A); Condition × Illumination Direction interaction: *F*(3, 24) = 0.18, *p* = 0.9. Overall, the sensitivity to changes in the blue chromatic direction was worst: Thresholds in the blue illumination-change direction were the highest and significantly higher than those in the green direction (*p* = 0.001; significant at a Bonferroni-adjusted significance level of *p* = 0.05/6 = 0.0083). Other across-direction comparisons did not reach the Bonferroni-adjusted significance level.

All statistical analyses carried out above conform to the plan described in the preregistration document for this experiment.

#### Note on Experiment 1A

**Figure A1:**
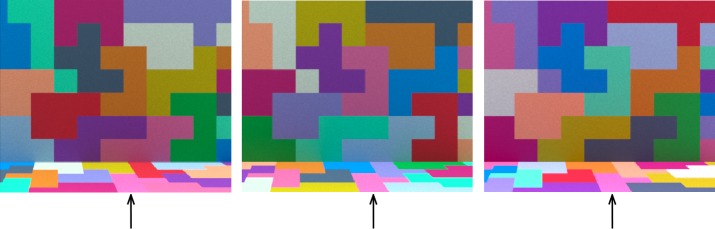
Examples of the stimuli in Experiment 1A. The target scene in the fixed-surfaces condition is shown on the left. Two different target scenes from the shuffled-surfaces conditions are shown in the center and on the right. In both conditions, the set of four pinkish tiles in the center of the floor (denoted with an arrow) remained fixed across all target and test images.

Experiment 1 reported above is a replication of Experiment 1A described in Appendix A. While preparing this work for publication we realized that due to a typo in the Blender file used for rendering of the stimulus images one of the surfaces (a subset of four tiles, Figure A1) in Experiment 1A was not under experimental control. Instead of the experimentally assigned surface reflectance, this surface was assigned a different pinkish-appearing (physically realistic) reflectance in the rendering pipeline. In both conditions, the reflectance of this surface remained fixed across all stimulus scenes. In other words, in the shuffled-surfaces condition of Experiment 1A the reflectance of all but this one surface varied across the stimulus scenes. For this reason, we conducted Experiment 1 as reported above, with the corrected set of rendered stimulus scenes. Comparison of the results of the two experiments, described in Appendix A, did not reveal any differences in the results. This indicates that the lack of experimental control over the one surface did not have a measurable effect on thresholds, a fact that is relevant to the interpretation of Experiment 2 below.

## Experiment 2

In Experiment 2 we aimed (a) to replicate the results of Experiment 1 with a different group of observers and a different set of stimulus scenes, and (b) to gain further insight into processing that underlies the illumination discrimination judgment by tracking observers' eye movements in the course of the task.

In Experiment 2, we were therefore able to compare observers' performance across fixed- and shuffled-surfaces condition not only in terms of thresholds, but also in terms of eye-fixation patterns. This allowed us to investigate whether observers systematically attend to a particular surface or a location in the scene and whether the pattern of fixations changes as a function of condition. Such differences could indicate that observers use different strategies to complete the illumination discrimination task when the surface reflectance layout changes across stimulus scenes versus when it remains fixed.

In Experiment 2 we used a different set of stimulus scenes from that used in Experiment 1, although the methods used to create a set of scenes in each condition were identical. The new set of scenes had the same geometry and used the same set of surface reflectance samples as the scenes in Experiment 1, but employed a different random tile-to-reflectance assignment. Unlike in Experiment 1, where the stimulus scenes were presented stereoscopically, in Experiment 2 the scenes were rendered from a single viewpoint, presented on a single screen and viewed monocularly. This change in viewing conditions should not affect our results: We have previously shown that eliminating stereoscopic information in experiments which use simulated scenes similar to those used here has no measurable effect on illumination discrimination thresholds (Radonjić et al., 2016, appendix A).

Experiment 2 was run after Experiment 1A and before Experiment 1, and was subject to the same artifact described for Experiment 1A (one surface did not vary in the shuffled-surfaces condition). Because we found no difference between the results of Experiments 1 and 1A, we did not rerun Experiment 2. In addition, the eye movement data from Experiment 2 indicate that observers rarely fixated the problematic surface: When averaged across observers, only 0.3% of all fixations in the experiment fall at this surface (from 0.02% to 0.9% across different observers).

### Methods

#### Preregistration

A document that describes the experimental design and the data analysis plan for this study was registered before the start of data collection. It is publically available at https://osf.io/x7hzu/ (main document) and https://osf.io/qg9cu/ (addendum).

#### Apparatus

Observers viewed the stimuli on a calibrated 24″ NEC PA241W color monitor driven at a pixel resolution of 1,920 × 1,200 and at a refresh rate of 60 Hz by a dual-port video card (NVIDIA GeForce GT 120; NVIDIA; and ATI Radeon HD 5770; AMD, Santa Clara, CA). The host computer was an Apple Macintosh with an Intel Xeon Quad-Core processor (Apple, Inc, Cupertino, CA).

We recorded the observers' eye movements using an eye tracker (EyeLink 1000, Desktop Mount, SR Research, Ottawa, Canada), driven by a host computer provided by SR Research (PC with Pentium Core Duo processor, using ROM-DOS Real-Time Operating System). The eye tracker was positioned in front and center of the LCD monitor (56 cm from the observer's eye) and configured for monocular tracking. Communication between the Eye Link and Apple host computer was accomplished using mgl routines written for this purpose, which relied on C code libraries provided by EyeLink. EyeLink recorded the position of the eye at the rate of 1,000 Hz.

In the experiment, the observer's head was stabilized using a chin rest, set so that the observer's eye height was approximately aligned with the center of the upper half of the monitor. The position of the chinrest was fixed across observers and sessions. The distance between the observer's eye and the center of the screen was 68.3 cm. In all observers we monitored the position of the right eye only while the left eye was covered with an eye patch.

#### Stimuli

We rendered a new set of stimulus scenes for Experiment 2. All scenes had the same geometry and used the same reflectance set for the tiles as in Experiment 1, but differed in their reflectance-to-tile assignment. In the fixed-surfaces condition this assignment was fixed across all stimulus scenes (but differed from that used in the fixed-surfaces condition of Experiment 1). In the shuffled-surfaces condition, reflectance-to-tile assignment was determined randomly at the time of rendering and differed for each stimulus scene (with an exception of one surface, as noted above). The experimental illuminations were identical to those used in Experiment 1.

All scenes were rendered from a single (cyclopean) viewpoint, as they were presented on a single screen. For each condition we rendered 30 target scenes (30 scenes × 1 target illumination) and 200 test scenes (1 scene × 50 illumination steps × 4 chromatic directions). The stimulus position of the screen was adjusted so that the stimulus scene (window into the simulated room) was in the center of the screen. At 68.3 cm viewing distance from the observer's eye, the stimulus image subtended 20° × 16.7° of visual angle (24.1 × 20.1 cm).

In the fixed-surfaces condition the mean luminance of the target scene was 17.25 cd/m^2^ (averaged across all target scenes), while the image chromaticity was *xy* = (0.336, 0.318; *SD* across scenes: 0.0015 for Y; < 0.0001 for *x* and *y*). In the shuffled-surfaces condition, mean luminance across target scenes was 17.42 cd/m^2^ (*SD*: 0.55) and mean chromaticity was *xy* = (0.327, 0.319), *SD*s (0.0048, 0.0046). The mean pairwise ΔE difference across all versions of the target image in the shuffled-surfaces condition was 3.20 (*SD*: 1.46; maximum: 7.30). These values were computed following the same methods as in Experiment 1.

#### Experimental procedures

The experimental procedures were identical to those in Experiment 1, apart from the differences due to introducing eye movement recordings. Because a different apparatus was used in Experiment 2, the presentation time of each stimulus interval was slightly larger than that of Experiment 1 (target interval: 2,370 ms; comparison interval: 870 ms; blank interval: 750 ms).

As in Experiment 1, at the beginning of each session the observers completed a training block that consisted of 12 trials and used the stimuli that corresponded to the condition run in that session. Eye movements were not recorded during the training.

At the beginning of each experimental block, we performed a 9-point calibration of the Eye Link, followed by a validation. During the calibration and validation, the screen background color was set to black (0.36 cd/m^2^). In most cases, we were able to obtain validations where the mean tracking error (difference between the actual and predicted fixation) across all tested points was equal to or less than 0.5° of visual angle and the largest tracking error at any single point did not exceed 1°. In the course of the experiment we relaxed this criterion slightly, allowing a mean error of up to 1° and a maximal error at the corner locations of up to 1.2°, as long as the validation was classified as “good” by the EyeLink software. The calibration at the corner locations was challenging because these areas fall outside of the EyeLink's optimal tracking range. These locations were also outside of the area of stimulus presentation.

After every 100 trials (approximately 10–15 min) the experimental block was paused to recalibrate the eye tracker and enable the observer to take a short break (∼5 min). The experiment continued after a successful calibration and its validation. An experimental session lasted between 60 and 90 min.

#### Observers

Ten observers participated in Experiment 2 (eight female, two male; age:19–22; none participated in Experiments 1 or 1A). Observers all had normal or corrected-to-normal visual acuity and color vision (as assessed via standard lab procedures described above). In addition, all observers passed an initial screening to ensure that we were able to reliably track their eye movements using EyeLink. This screening consisted of a 10–15 min long session in which we performed a series of eye-tracking calibrations and validations (for the right eye only). We enrolled only observers who successfully completed several successive 9-point calibrations and corresponding validations (mean tracking error of 0.5° or less; maximal tracking error of 1° or less).

Three observers who passed all the initial screenings (all female, age: 20–22) withdrew from the study during or after the first session citing fatigue and difficulty of keeping still for a prolonged period of time. Data from these observers were not analyzed.

#### Data analysis method

Thresholds are computed from the data following the same procedures as in Experiment 1. In line with the preregistration document for this experiment, the main analysis of observers' threshold measurements was done using a two-way repeated measures analysis of variance (ANOVA) with condition (fixed vs. shuffled surfaces) and illumination-change direction (blue, green, red, and yellow) as fixed within-subject factors.

For the purposes of eye movement analysis, for each interval of each trial in the experiment, we extracted fixations from the recorded eye positions via a velocity-based algorithm developed by Nyström and Holmqvist (2010). This algorithm uses an adaptive method, based on the data in a single trial interval, to detect onset and offset of saccades within that interval. The Nyström/Holmqvist algorithm has several free parameters. In our implementation, parameters were set as recommended in the published version of the code (http://dev.humlab.lu.se/www-transfer/people/marcus-nystrom/EventDetector1.0.rar), except for the following two: The initial saccade peak velocity detection threshold was set to 80 (following Boghen, Troost, Daroff, Dell'Osso, & Birkett, 1974; Bahill, Clark, & Stark, 1975), and the minimal fixation duration was set to 100 ms, a value more appropriate for tasks involving scene perception (Nyström & Holmqvist, 2010; Salvucci & Goldberg, 2000). Because of its adaptive nature, the Nyström/Holmqvist algorithm successfully detects fixations that the built-in EyeLink fixation detection algorithm misses, in particular those that occur when observers move their eyes minimally within an interval.

#### Exclusion criteria based on missing eye movement data

There were times when eye position was not recorded (due to observer's blinking or technical difficulties that caused EyeLink to lose track of the eye position) or when the recorded eye position fell outside of the display boundaries. For each trial, we computed the proportion of such data during each of the three intervals and excluded from further analysis trials for which this proportion exceeded 5% in any of the intervals. We also excluded any trial for which the Nyström/Holmqvist algorithm either (a) identified that more than 20% of data points were blinks or noise in any of the intervals or (b) did not detect a fixation within the display boundary in one or more of the stimulus intervals. In addition, we also excluded from the analysis 69 trials for one observer (EOM; 2nd session in the fixed-surfaces condition) because, after a break, the experiment was continued without acquiring new calibration and validation of the eye position. Based on all these criteria, we excluded 20.3% of trials per observer on average. Across observers, the mean proportion of excluded trials (averaged across sessions) ranged from 4.4% to 39.4%. There was no systematic change in the proportion of excluded trials across the four sessions, *F*(3, 27) = 2.43, *p* = 0.09. A table showing the total number of excluded trials for each observer and condition is available in the online supplement. Note that these exclusions apply only to the eye fixation analysis (all trials are included in the threshold analysis).

To mitigate against the possibility that the Nyström/Holmqvist algorithm over-detected fixations, we filtered the identified fixation locations by removing any that were less than 1° of visual angle away from the preceding identified fixation location. Before filtering, we also excluded all fixations that fell outside of the display boundaries. These two steps reduced the total number of fixations by 8.16% on average (between 0.8% and 20.2% of fixations removed per observer). All eye fixation analyses we report below are based on the filtered fixations. For each observer, we provide the raw eye position data in the online supplement.

### Results

#### Illumination discrimination thresholds

**Figure 4:**
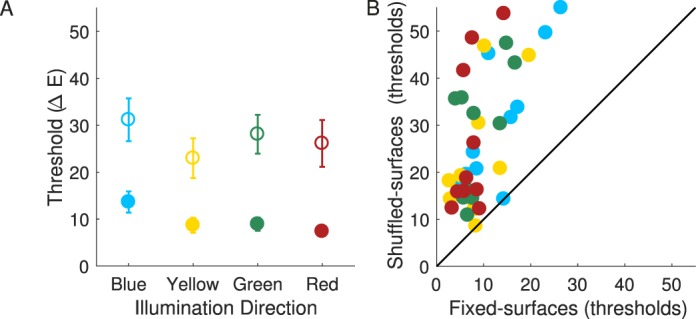
Experiment 2 Results. Panel A. Mean thresholds in the fixed-surfaces (filled circles) and shuffled-surfaces condition (open circles). Panel B. Individual observers' thresholds in the shuffled-surfaces condition are plotted against those in the fixed-surfaces condition for each illumination-change direction. The figure panels follow the same notation conventions as panels in Figure 3.

The results of Experiment 2 replicated those of Experiment 1 (Figure 4). All observers were able to complete the illumination discrimination task in the shuffled-surfaces condition (thresholds in each illumination-change direction were within the stimulus range in at least one session). For four of these observers the thresholds fell outside of the stimulus range in one session for some illumination-change directions: one for two different observers (blue for one, green for another) and two for two remaining observers (blue and yellow for one, and blue and red for the other).

As in Experiment 1, the sensitivity to illumination changes in the shuffled-surfaces condition was considerably worse than in the fixed-surfaces condition: main effect of condition, *F*(1, 9) = 32.76, *p* < 0.001. We compared individual observers' thresholds across conditions for all illumination-change directions (Figure 4B) and found only two instances (out of 40) in which the thresholds across the two conditions were essentially the same (one instance each for two different observers). The mean thresholds in the shuffled-surfaces condition were 27.1 ΔE, as compared to 9.7 ΔE in the fixed-surfaces condition—a 17.4 ΔE increase. The degree to which overall thresholds increased in the shuffled-surfaces condition varied across observers (from 8.3 to 33.7 ΔE when averaged across illumination directions). As in Experiment 1, this increase in thresholds is considerably larger than the estimated variation in the image mean due to shuffling (less than 3.5 ΔE; see Experiment 2 Methods).

As in Experiment 1, thresholds differed significantly across the illumination-change directions: main effect of direction, *F*(3, 27) = 6.37, *p* < 0.005, and the pattern of threshold variation across illumination directions was similar in the two conditions: Condition × Illumination Direction interaction was not significant, *F*(3, 27) = 0.86, *p* = 0.5. Thresholds in the blue illumination-change direction were again the highest and significantly higher than for either the yellow (*p* < 0.005) or red (*p* < 0.005) directions. No other paired comparisons were significant after Bonferroni correction.

We conducted a posthoc analysis (not included in the preregistered analysis plan) to explore whether there were individual differences in the degree to which a variation in the reflectance layout affected performance. As in Experiment 1, an ANOVA with observer modeled as a random factor revealed an Observer × Condition interaction, *F*(9, 79) = 6.47, *p* < 0.001; main effect of observer or any other interactions across factors were not significant.

#### Distribution of fixations across intervals and conditions

To gain additional insight, we analyzed the pattern of observers' eye fixations. As noted in the preregistration document, the eye-movements analyses we conducted are exploratory.

**Figure 5:**
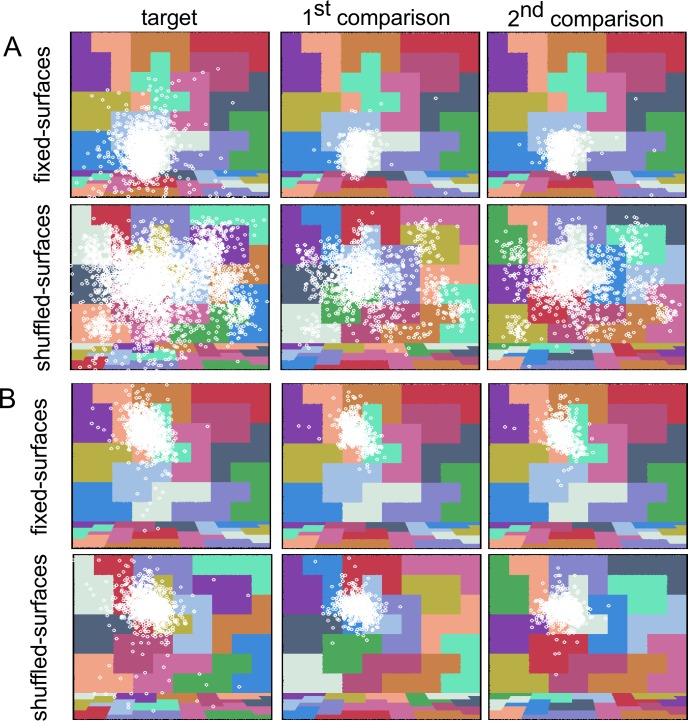
Eye fixation distributions for two sample observers. Panel A: observer AZM. Panel B: observer EOM. For each condition and interval fixations are aggregated across all trials and overlaid onto the corresponding locations in the stimulus image (white circles). Top row: fixed-surfaces condition. Bottom row: shuffled-surfaces condition. First column: target interval. Second column: first comparison interval. Third column: second comparison interval. The images shown are cropped so that only the stimulus image is shown. Figures showing fixations over the entire display (including areas outside of the image) for each observer are available in the online supplement. Tile colors shown in this figure are for illustration purposes only. A tone-mapped rendering of the stimulus is shown in Figure 1.

Figure 5 shows the fixation distributions for each condition for two of our observers. For each interval, the fixations are aggregated over all trials and plotted onto corresponding locations of the stimulus image. Two key characteristics of observers' fixation patterns are illustrated by this figure and confirmed in the quantitative analyses we present below.

First, the distribution of fixations across the three intervals within a condition is quite similar within observer. That is, each observer tends to look at the same general stimulus locations across intervals, although in some cases the spread of fixations in the target interval, which was longer, is larger than for the comparison intervals. Across observers, however, which particular stimulus locations are looked at can vary. Figures in the same format as Figure 5 for all observers are available in the online supplement.

Second, there are individual differences in how fixation patterns differ between conditions. For some observers, the fixation distribution changes considerably—from more concentrated in the fixed-surfaces condition to more diffuse in the shuffled-surfaces condition (Observer AZM, Figure 5A). For other observers the fixation patterns are similar across conditions. While some of these observers tend to look at a fairly small region of the stimulus image in both conditions (Observer EOM, Figure 5B), for others, fixations are distributed widely across the entire stimulus image independent of condition (Observer DTM, see online supplement).

To quantify the patterns in the fixation data, we analyzed the number and distribution of observers' fixations across conditions as well as the observers' tendency to look at surfaces that have the same reflectance across intervals of a single trial. To understand the relationship between subject performance and the fixation patterns, we then investigated whether any of the fixation characteristics we analyzed predicted thresholds.

**Figure 6:**
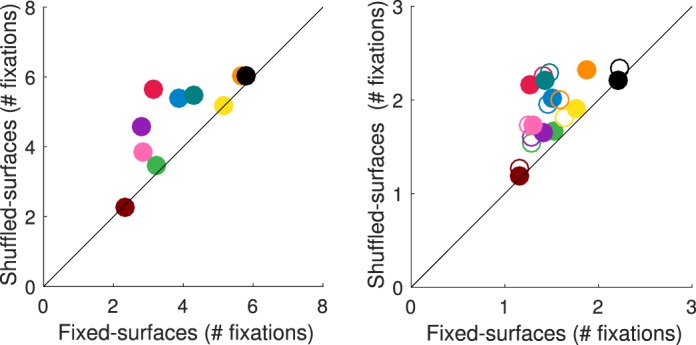
Number of fixations across conditions. For each observer the average number of fixations (across trials) in the shuffled-surfaces condition is plotted against the corresponding average in the fixed-surfaces condition (left panel: target interval; right panel: comparison intervals; filled circles indicate first comparison interval, and open circles second comparison interval). The standard error of the measurement (±1 SEM) is in all cases smaller than the plotted points. A unique color is used for each observer. Observer AZM (Figure 5A) is shown in red and observer EOM (Figure 5B) in brown. In the supplement, a key is provided to relate each observer's fixation plots to the color code scheme used in this and similar figures below.

Figure 6 shows the average number of fixations for the shuffled-surfaces condition versus the fixed-surfaces condition for each observer (left panel: target interval; right panel: comparison intervals). The majority of data points fall above the diagonal, showing that some (but not all) observers made more fixations in the shuffled- than in the fixed-surfaces condition.

We further investigated this observation via a repeated measures ANOVA with condition (shuffled- vs. fixed-surfaces) and interval (target vs. comparison) as fixed factors and observer as a random factor. As the mean number of fixations across the two comparison intervals did not differ significantly in either condition: fixed-surfaces, *t*(9) = 1.6, *p =* 0.14; shuffled-surfaces, *t*(9) = 0.7; *p* = 0.5, these data were combined in the ANOVA by averaging across intervals. The ANOVA confirmed that observers made more fixations in the shuffled-surfaces condition than in the fixed-surfaces condition, *F*(1, 9) = 13.17, *p* < 0.01. As expected, all observers made fewer fixations in the comparison intervals, which were considerably shorter than the target interval, *F*(1, 9) = 87.5, *p* < 0.001, but the size of this effect varied both across conditions and across observers: Condition × Interval interaction: *F* (1, 9) = 5.51, *p* < 0.05; Observer × Interval interaction, *F* (9, 39) = 7.31, *p* < 0.01. We did not find a significant main effect of observer or Observer × Condition interaction.

**Figure 7:**
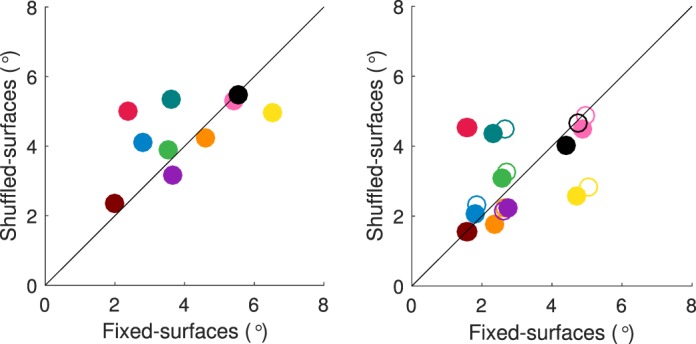
Spread of fixations across conditions. The joint standard deviation of fixation locations in the shuffled-surfaces condition is plotted against the joint standard deviation (in degrees of visual angle) in the fixed-surfaces condition for each observer (left panel: target interval; right panel: comparison intervals with filled circles indicating the first, and open circles the second comparison interval). Colors indicate observers, using the same scheme as in Figure 6.

We also quantified the spread of observers' fixations. For each observer and condition we first aggregated the fixation positions (expressed as *x* and *y* screen coordinates, in degrees of visual angle) of all fixations in a given interval across trials and computed the standard deviation for the *x* and *y* coordinates separately. We then computed a single joint standard deviation, as the square root of the sum of squared *x* and *y* standard deviations. In Figure 7, we compare the spread of fixations, computed in this way, for the shuffled-surfaces versus the fixed-surfaces condition for each observer (left panel: target interval; right panel: comparison intervals). Across observers, data deviate from the diagonal in both directions, suggesting that some observers had a wider spread of fixations in the shuffled-surfaces condition, while others showed no condition effect or (for one observer) an effect in the opposite direction.

We confirmed this observation via a repeated measures ANOVA with condition (shuffled- vs. fixed-surfaces) and interval (target, first comparison, and second comparison) as fixed-factors and observer as a random factor. Although the main effects of condition or observer were not significant, *F*(1, 9) = 0.4, *p* = 0.5; *F*(9, 59) = 2.3, *p* = 0.1, respectively, we found a significant Observer × Condition interaction, *F*(9, 59) = 94.15, *p* < 0.001. The spread of fixations was the largest in the target interval and smallest in the second comparison interval: main effect of interval, *F*(1.1, 10) = 37.6, *p* < 0.001; across-interval pairwise comparisons are all significant at the *α* = 0.05 level, after Bonferroni correction. Also, the degree of difference in fixation spread across intervals varied across observers: Observer × Interval interaction, *F*(10, 32.7) = 8.08, *p* < 0.001.

**Figure 8:**
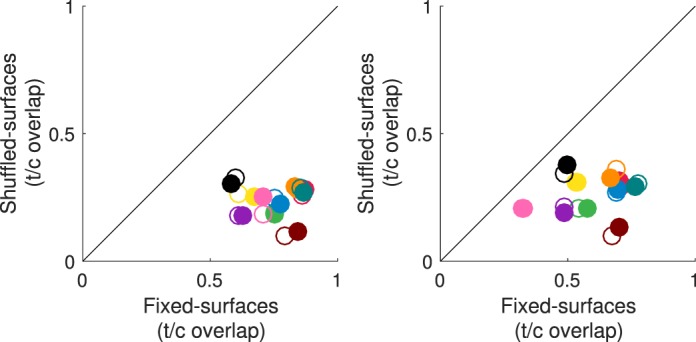
Surface reflectance fixation analysis. Left panel. The proportion of unique surface reflectances fixated in the comparison interval that overlap with those from the target interval (averaged across trials) is compared across the shuffled-surfaces condition (y axis) and fixed-surfaces conditions (x axis; label “t/c overlap” refers to target/comparison overlap). Filled circles: target versus first comparison interval; Open circles: target versus second comparison interval. Colors indicate observers, using the same scheme as in Figure 6. SEM (±1) is in all cases smaller than the plotted points. Right panel. Same as left panel, but where each target interval is paired with comparison intervals chosen at random from different trials.

Finally, we examined the pattern of observers' eye movements across the intervals of an illumination discrimination trial. Specifically, we asked whether observers tend to fixate on the same surface reflectance (or set of surface reflectances) across trial intervals. Discriminating changes in illumination by fixating on surfaces with the same reflectance could be a fruitful strategy. In the fixed-surfaces condition, this would only require looking at the same location across intervals. In the shuffled-surfaces condition, however, implementing this strategy would be challenging, as it would require fixating on different image locations across intervals, in a manner that compensated for the shuffling. In other words, the observer would have to quickly locate and attend to the tracked reflectance type, which is in a novel location on each trial interval.

To investigate this condition, we computed the proportion of unique surface reflectances fixated on in each comparison interval that were also fixated on in the target interval. For each observer and condition, the left panel in Figure 8 shows this proportion in the shuffled-surfaces versus the fixed-surfaces condition (filled symbols indicate first comparison interval; open symbols indicate second comparison interval). For all observers, this number is considerably lower in the shuffled-surfaces condition, indicating that fixations on the same surface reflectance across intervals are significantly reduced: paired *t* tests, *t*(9) = 13.49; *p* < 0.001, for the target versus first and *t*(9) = 12.72; *p* < 0.001 for the target versus second comparison interval. To determine whether the number of trials where the observers looked at the same surface reflectance across intervals was greater than one would expect based on their aggregate (across trials) fixation behavior, we recomputed the same statistic but with fixation data for each comparison interval shuffled across trials. A different shuffling was used for each of the two comparison intervals. The results are shown in the right panel of Figure 8. For the fixed-surfaces condition, trial-shuffling reduces the proportion of surface reflectance overlap in the fixed-surfaces condition, but the numbers remain above zero. This suggests that observers tend to fixate on the same (subset of) surface reflectances across trials. For the shuffled-surfaces condition, however, trial-shuffling does not change the numbers much, suggesting that in this condition the overlap in fixated surface reflectances across intervals is already close to what would be achieved by chance.

In a complementary analysis we quantified the degree of fixation location overlap, rather than surface reflectance overlap. The results of this analysis (presented in Appendix B) show that locations of fixations also overlap substantially across trial intervals (although the degree of overlap varies across observers) and remains above zero when the target and comparison intervals are paired randomly across trials. This suggests that in the fixed-surfaces condition, our data cannot distinguish between two possible underlying strategies employed by observers: (a) a tendency to fixate on the same locations across intervals (leading to fixating on surfaces with the same reflectance) or (b) a tendency to fixate on surfaces with the same reflectance (leading to fixating on the same stimulus locations).

**Figure 9:**
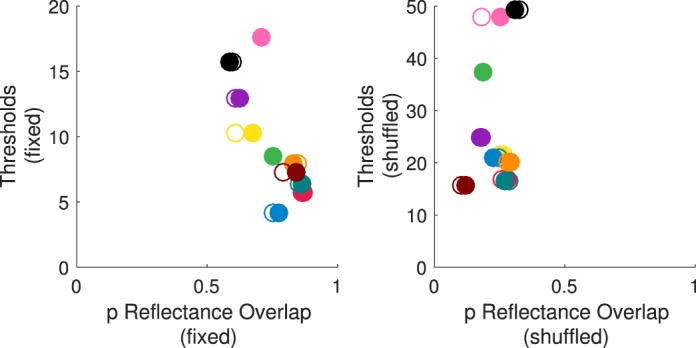
Surface reflectance overlap predicts illumination discrimination thresholds in the fixed-surfaces but not in the shuffled-surfaces conditions. Left panel: fixed-surfaces condition. Right panel: shuffled-surfaces condition. For each observer the average illumination discrimination threshold is plotted against the proportion of fixations in the comparison intervals that overlaps with those from the target interval (filled circles: target vs. first comparison interval; open circles: target vs. second comparison interval). The difference in y axis scale between the two panels is due to a different threshold range for the fixed- versus shuffled-surface condition.

We assessed whether the degree to which observers fixate on common surface reflectances across trial intervals predicts performance in the illumination discrimination task. Figure 9 compares the proportion of surface reflectance overlap (Figure 8, left) with mean observer threshold in each condition. In the fixed-surfaces condition, the two quantities were related: The higher the degree of surface overlap, the lower the illumination discrimination threshold: Spearman's rank-order correlations, *r*(8) = −0.81, *p* < 0.01, for target and first comparison intervals; *r*(8) = −0.72, *p* < 0.05 for the second comparison interval. In contrast, we found no significant correlation between the thresholds and fixation overlap in the shuffled-surfaces condition.

A similar analysis relating the performance and the characteristics of the fixation pattern revealed that in the fixed-surfaces condition, both a low fixation spread and a high fixation location overlap across trials were also predictive of better performance in the illumination discrimination task, while there was no systematic relationship between performance and the number of fixations. Interestingly, none of the quantities we measured were predictive of observers' performance in the shuffled-surfaces condition. A detailed description of these analyses is available in Appendix B.

Finally, we also examined the relative distribution of fixations across different surface reflectances in the stimulus scene across conditions. For each observer we aggregated the data about the surface reflectance at each fixation (for each interval and condition) across all trials and plotted the relative proportion of fixations for each reflectance. The plots are available in the online supplement. For the majority of our observers, the fixation distributions in the fixed-surfaces condition had between one and three peaks, indicating the tendency to fixate predominantly on a few reflectance samples. Interestingly, observers tend to favor similar reflectances. Across our ten observers, the two most often fixated reflectances were ones that appeared pale-green (turquoise) and pale-blue (6/20 each), followed by ones that appeared light-gray (3/20) and pale-orange (2–4/20, depending on the interval). These four reflectances, which account for 88% of three most-fixated reflectances across observers and intervals, were centrally located in the stimulus scene (middle of the back wall). They were also the four highest luminance surfaces in the scene. This result is generally consistent with the findings showing that observers tend to look at the brightest regions of the object when asked to judge its lightness (Toscani, Valsecchi, & Gegenfurtner, 2013; but see also te Pas et al., 2017). Other possible factors contributing to the high frequency of fixations at these surfaces might be their central location and the fact that these, less saturated surfaces, more closely resemble the reflectances typically found in natural scenes (Krinov, 1953; Vrhel, Gershon, & Iwan, 1994). In the shuffled-surfaces condition the distribution of fixations per reflectance sample was approximately flat for all observers. Within a condition, differences across intervals (target, first vs. second comparison interval) were negligible.

### Discussion

#### Effect of surface shuffling on thresholds

We investigated whether sensitivity to changes in illumination is affected by the stability of the surface reflectance layout in the scene. To this end, we conducted two experiments in which we compared observers' performance across conditions in which the surface reflectance layout was fixed across stimulus scenes and where it varied as the illumination changed. In the first experiment, we investigated only the effects of dynamic surface changes on thresholds. In the second experiment, we also measured eye movements to assess whether differences in eye-fixation patterns were predictive of differences in performance or informative about task strategies, both across observers and conditions. The two experiments used different observers and slightly different stimuli, but the results were essentially identical. When the surfaces in the scene remained fixed, illumination discrimination was good and performance was similar to that measured in our previous studies (Pearce et al., 2014; Radonjić et al., 2016). When the reflectance layout varied, observers' performance was considerably impaired, even though the surface reflectance shuffling preserved (to a good approximation) the mean of the stimulus images: Thresholds in the shuffled-surfaces condition increased by 16.5 ΔE in Experiment 1 and by 17.4 ΔE in Experiment 2. This large increase in thresholds cannot be accounted for by the slight variation in image mean due to shuffling, which we estimate to be less than 3.6 ΔE on average. Although performance was impaired, discrimination of changes in illumination was still possible in the shuffled-surfaces condition: For all but one (out of 30) observers we tested across Experiments 1, 1A, and 2, performance was significantly better than that of a randomly responding observer.

Our results speak against the hypothesis that observers use only the information from global scene averages to discriminate changes in scene illumination. Indeed, even when the mean reflectance of the images is held fixed, shuffling the assignment of reflectances to surfaces has a dramatic effect on illumination discrimination thresholds. To put it another way, in our experiments fine discrimination of illumination changes requires that the surface reflectance at each location in the scene remains fixed.

Our results are in agreement with those of Foster and colleagues (Craven & Foster, 1992; Foster & Nascimento, 1994), who measured observers' ability to discriminate changes in scene illumination from changes in material of surfaces in the scene. When the cone-excitation ratios at edges varied, as in our shuffled-surfaces condition, observers are more likely to interpret the changes in the scene as changes in surface material, rather than changes in illumination, in the absence of a salient global change in the image mean. Thus, it is possible that in our shuffled-surfaces condition, what is perceived as changes in surface reflectances masks the perception of the illumination change. Our results also connect with work that manipulates the spatial structure of chromatic backgrounds/textures as a way of probing how the visual system parses image variation to underlying variation in either illumination or surface reflectance (Schirillo & Shevell, 2000; te Pas & Koenderink, 2004; Zaidi, Spehar, & DeBonet, 1997).

The shuffling manipulation does not completely destroy observers' ability to detect changes in illumination: Measured performance in the shuffled-surfaces condition was significantly better than what would be expected from an observer who responded randomly. This result shows that observers are able to make a coarse judgment of illumination change in the absence of a fixed surface layout. An open question is whether the level of performance observed in this condition is the result of mechanisms that integrate information across surfaces within each stimulus interval to extract an illumination estimate, the result of comparing low-level signals across the locations fixated in each stimulus interval, or a combination of both. More generally, how the visual system integrates local and global information to perform the illumination discrimination task remains an open question that requires more focused experimentation.

In our experiment, we varied the surfaces in the stimulus scene by shuffling the reflectances while keeping the scene surface layout fixed. This offered us a simple way to randomize surface reflectance assignment in the scene while keeping the mean reflectance roughly constant. It is also possible to vary the layout of surfaces in the scene in a manner that does not preserve the underlying scene geometry. We have not explored this class of variations. Therefore, how variations in geometric layout interact with variations in surface reflectance assignment to influence illumination discrimination also remains an open question. Our results show, however, that varying the surface reflectance assignment alone is sufficient to produce large elevations of illumination discrimination thresholds.

Another open question concerns the choice of surfaces reflectances we used. In our studies of illumination discrimination to date, the reflectances used to tile the stimulus scenes produced fairly saturated colors, which are less typical in natural viewing (Krinov, 1953; Vrhel, Gershon, & Iwan, 1994). We previously showed that illumination discrimination does depend on specific characteristics of the scene reflectance ensemble (Radonjić et al., 2016), and it is possible that, overall, discrimination would be different in scenes that employ more naturalistic surface reflectances. It is unlikely, however, that the choice of reflectances would considerably modulate the large effect of surface-reflectance shuffling we report here.

Note also that because in the fixed-surfaces condition the surface reflectance layout remains fixed across all trials, we cannot rule out the possibility that some degree of learning of that layout occurred due to repeated exposure, and that this facilitated the discrimination of illumination changes in this condition. If this were the case, our measures would provide a lower bound on illumination discrimination thresholds in the fixed-surfaces condition. Future research could address this question by directly comparing illumination discrimination when the reflectance layout remains fixed across intervals but varies across trials.

#### Threshold variation across chromatic directions

In our previous studies, which probed illumination discrimination using scenes in which the surfaces remained fixed across stimulus intervals, we consistently found that sensitivity to changes in illumination varies across different chromatic directions when the CIELUV metric is used to quantify the magnitude of the physical illumination change (Pearce et al., 2014; Radonjić et al., 2016). Here we show that this result generalizes to scenes in which the surface reflectance layout and illumination are varied concurrently. In both experiments, the pattern of threshold variation was similar in the fixed- and shuffled-surfaces condition (but see Experiment 1A). Overall, sensitivity was the worst for the blue direction—a result we have obtained consistently both in this study and previous studies in which the ensemble of surface reflectances in the scene was roughly neutral (Pearce et al., 2014; Radonjić et al., 2016). Across experiments, we did find some differences in results of statistical tests that compared, pairwise, the thresholds across different directions (see Results). We have reported similar differences across well-matched experiments before (Radonjić et al., 2016). The consistent across-experiment replication of the finding that sensitivity to illumination changes in the blue chromatic direction is the lowest represents, we believe, the strongest evidence that this is a real characteristic of human vision. Definitive statements about the relative ordering of thresholds in the yellow, green, and red directions are harder to make, because this ordering has varied across our experiments. Such variations might occur because the differences are consistent but small enough that we do not have sufficient experimental power to reliably characterize them or because the relative ordering of thresholds in these directions depends on small variations in experimental design which we have not identified or systematically controlled.

#### Insights from the analyses of eye-fixations

In Experiment 2, we recorded eye fixations as observers performed the illumination discrimination task. Previous work has shown that the pattern of observers' fixations depends on task goals (Ballard, Hayhoe, & Pelz, 1995; Hayhoe & Ballard, 2005) and can be optimized to extract the information required to complete a task (Najemnik & Geisler, 2005). Both where observers look as they perform a task and how they adapt the fixation patterns as task demands change provide insight into what information observers use to complete the task.

Our analysis of fixation patterns reveals that in the fixed-surfaces condition three characteristics predict good overall illumination discrimination: (a) a tendency to fixate on the same surface reflectance across trial intervals, (b) a small overall fixation spread, and (c) a high overlap of fixation locations across trial intervals. Fixation patterns with these characteristics are consistent with an observer strategy that involves looking at the same image location across intervals in the illumination discrimination task. In the fixed-surfaces condition this strategy also has the effect that observers look at surfaces with the same reflectances across intervals. Thus, in the fixed-surfaces condition, the two strategies—looking at the same stimulus locations versus looking at the same subset of surface reflectances—cannot be distinguished from each other based on our data.

Although the task demands were different in the shuffled-surfaces condition, observers' fixation patterns showed little change: Overall, the observers did make more fixations, but this effect was not large. Furthermore, analysis of the fixation locations across trial intervals (as well as across trials) suggests that even in the shuffled-surfaces condition, observers look at the same general area of the stimulus scene at higher-than-chance levels. This might reflect a carry-over effect of a strategy that was effective in the fixed-surfaces condition but not in the shuffled-surfaces condition. The effect of this tendency in the shuffled-surfaces condition is that observers were considerably less likely to fixate surfaces with the same reflectance across intervals. We did not, however, find any individual observer characteristics of fixations in the shuffled-surfaces condition that predicted performance in that condition.

**Figure 10:**
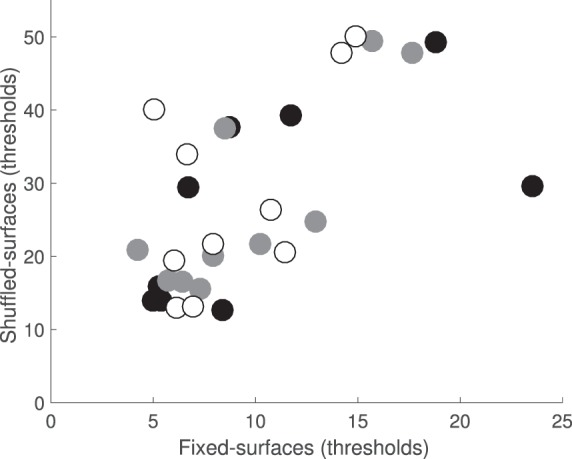
Thresholds in the fixed-surfaces condition predict thresholds in the shuffled-surfaces condition. Thresholds, averaged across illumination-change directions, are plotted for each observer who successfully completed the illumination discrimination task in both conditions (N = 29). Black circles: Experiment 1. Gray circles: Experiment 2. White circles: Experiment 1A.

Interestingly, we did find that observers' performance in the fixed-surfaces condition predicted their performance in the shuffled-surfaces condition. This is illustrated by Figure 10 in which mean thresholds (averaged across illumination directions) in the shuffled-surfaces condition are plotted against those in the fixed-surfaces condition for all 29 observers who successfully completed the illumination discrimination task (Experiment 1 is shown in black symbols, Experiment 2 in gray, and Experiment 1A white). The correlation of thresholds across conditions, computed cumulatively across observers was significant: Pearson's *r*(27) = 0.64, *p* < 0.001. Thus, some factor that causes observers to have low thresholds does generalize across conditions.

#### Observers' self-described strategies

To gain additional insight into potential strategies that observers used, at the end of the experiment we asked observers to describe, in their own words, how they completed the illumination discrimination tasks. The full set of responses is provided in the online supplement. An informal analysis indicates that most of the responses fell into one of the three categories. Some observers talked about a tendency to track an individual surface or group of surfaces across the trial intervals (often the lightest ones), e.g., “I used the white part of the room to determine which light matched better. The white blocks changed color most noticeably” (observer 8500, Experiment 1A). Some indicated trying to extract global information about the illumination from the scene, e.g., “I tried to let my eyes unfocus a bit to get a general impression of the lights as a whole” (observer VVU, Experiment 2). However, the majority of observers reported that they used a mixture of these two strategies, often favoring surface-tracking in the fixed-surfaces condition and global estimates in the shuffled-surfaces condition. For example:

*For the 1st and 3rd sessions (where the image on the screen was constant), I would focus my attention on the single-colored block, most often the whitish one. That would allow me to notice any slight alteration in the tone. For 2nd and 4th session I found the task to be easier if I would squint plus focus my attention on the general quality of light as a whole. This prevented me from becoming distracted when the colored blocks would change from scene to scene* (observer 10300, Experiment 1);

*For Experiments 2 and 4 (shuffled-surfaces) I had to concentrate on the bigger picture…whereas for 1 and 3 (fixed-surfaces) I only looked at the white spot at the upper right hand corner* (observer 8200, Experiment 1A).

We were unable, however, to identify any clear correspondence between observers' introspective comments and their performance and eye fixation characteristics.

#### Further directions

As a complement to the results presented in the current paper, we have begun to analyze performance for an idealized computational observer in the fixed-surfaces condition of our illumination discrimination task. The computational observer makes optimal use of the signals carried by the cone mosaic, where performance is limited by the Poisson noise of photoisomerization. Our initial results indicate that there is no clear relation between the pattern of human and computational observer's performance, thus implicating postreceptoral mechanisms as playing a key role in the chromatic illumination discrimination task. A full description of this modeling work is presented in a separate publication (Ding et al., 2018). More generally, understanding what stimulus information observers rely on to perform the task in both the fixed- and shuffled-surfaces conditions, as well as how this information is extracted from early visual representations, is of considerable interest.

Many questions about the processes that mediate performance in the illumination discrimination task remain open. For example, the relation between the mechanisms that underlie performance in this task and those that have been elucidated by classic studies of chromatic discrimination for spatially simple stimuli (Eskew, 2008; Stockman & Brainard, 2010) is still not well understood. In addition, the stimuli in our task are presented sequentially, which suggests that short-term memory might play a role in performance. Both of these aspects are topics of current investigation, both by our group (Aston, 2017) and others (Weiss & Gegenfurtner, 2016).

#### Concluding remarks

There are three complementary reasons that motivate our interest in understanding illumination discrimination.

First, in natural viewing illumination provides useful information in and of itself. For example, both time of day and upcoming weather are related to the spectrum of the illumination (Spitschan et al., 2016, provide data in support of the former; our assertion about the latter is anecdotal). How precisely observers encode and represent illumination thus tells us about a fundamental and useful perceptual ability, and our results add to the growing body of experimental literature on this question. In natural viewing, there are both situations where one would want to estimate illumination when the surfaces in the scene remain fixed (e.g., to discriminate illumination changes that occur over time in the same general environment) and where one would want to estimate illumination when the surfaces are not known (e.g., when stepping outside into an unfamiliar location). Our results suggest that sensitivity to illumination changes depends on the degree to which the surface reflectance assignment in the scene is held fixed, even in the restricted case where the mean reflectance remains constant. This conclusion reinforces that from our earlier work (Radonjić et al., 2016), in which we showed that even when the surfaces are held fixed, discrimination performance depends on the specific ensemble of surface reflectances in the scene. Theories of illumination discrimination should explicitly incorporate these findings.

Second, illumination discrimination may be predictive of the constancy of perceived object surface color (Álvaro et al., 2017; Pearce et al., 2014). The idea behind this hypothesis is that the higher illumination discrimination thresholds, the more stable the color appearance of the surfaces in the scene. On the other hand, the link is not a necessary one—the representations underlying illumination discrimination could be different from those that mediate object color appearance (see Weiss, Witzel, & Gegenfurtner, 2017). If illumination discrimination is predictive of object color constancy, then the fact that discrimination thresholds substantially increase when the scene surfaces are shuffled would lead to the prediction that the constancy of surface color appearance would increase considerably under the same conditions. A good test of this prediction would require an experimental paradigm where the observer was clear on which surface should be judged across a change of illumination as the surfaces in the scene were shuffled across that same illumination change. Providing a distinct cue to the identity of surfaces which relocate spatially under a concurrent change in illumination, to test the constancy of their appearance, might also provide a cue that increases color constancy. Nonetheless, the results of a controlled test of the prediction has the potential either to support or place limits on the link between surface appearance constancy and illumination discrimination.

Third, illumination discrimination provides an opportunity for generalizing our understanding of psychophysical discrimination beyond highly simplified laboratory stimuli, in particular to ask about the precision of encoding of distal stimulus variables. In the illumination discrimination experiment, observers are free to fixate different image locations across different stimulus intervals, and we can manipulate where in the image information is carried. Although we are far from a formal theory of performance in the illumination discrimination task, both the deleterious effect of surface-shuffling and the measured characteristics of eye fixations provide initial data about how observers perform the task. In particular, it appears that observers use information from fairly localized image regions, at least when placed under the time constraints of our three-interval forced-choice design. In this context, our current work demonstrates that systematic manipulation of physical factors in our stimulus scenes (such as scene illumination and surface reflectance assignment) can provide insight into the cues human observers use as they form perceptual representations of distal stimulus variables.

## Figures and Tables

**Figure A2:**
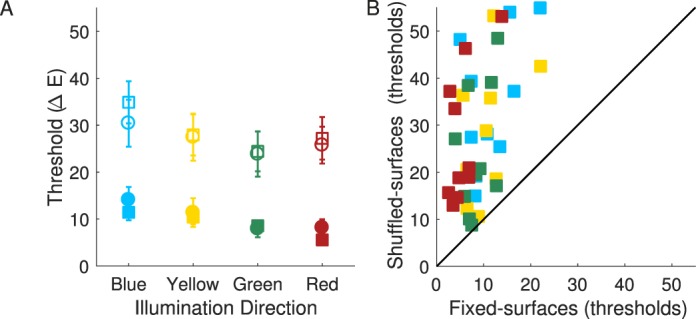
Experiment 1A Results. Panel A. Mean illumination discrimination thresholds (in ΔE, averaged over observers) for the fixed-surfaces (filled squares) and shuffled-surfaces condition (open squares) are shown for four chromatic directions of illumination change. Error-bars indicate ±1 SEM. Results of Experiment 1 are shown for comparison using circles (filled: fixed-surfaces; open: shuffled-surfaces condition). Panel B. For each observer and illumination-change direction, thresholds in the shuffled-surfaces condition are plotted against thresholds in the fixed-surfaces condition (symbol colors correspond to illumination-change direction).

**Figure B1:**
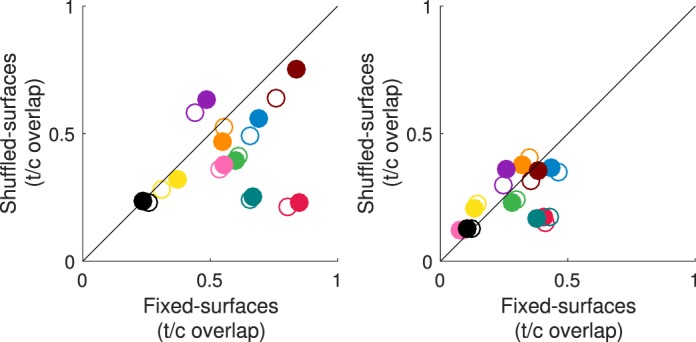
Overlapping fixation locations across intervals. Left panel: Mean proportion of fixations in the comparison interval that overlap with fixations from the target interval. Filled circles: target versus first comparison interval. Open circles: target versus second comparison interval. Right panel. Same as left panel, but where each target interval is paired with comparison intervals chosen at random from different trials. Colors indicate observers, using the same scheme as in Figure 6. SEM (±1) is in all cases smaller than the plotted points.

**Figure B2:**
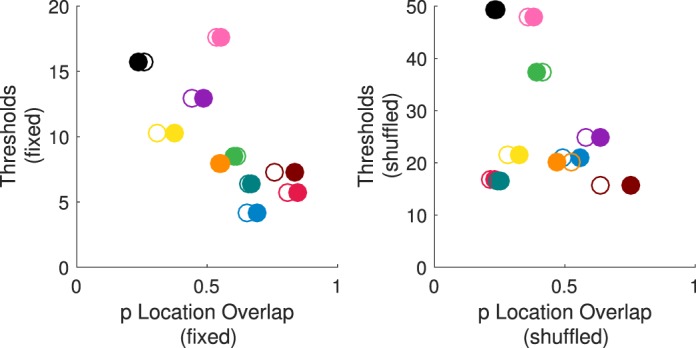
Fixation location overlap predicts illumination discrimination thresholds in the fixed-surfaces but not in the shuffled-surfaces conditions. Left panel: fixed-surfaces condition. Right panel: shuffled-surfaces condition. For each observer the average illumination discrimination threshold is plotted against the proportion of fixations in the comparison intervals that overlaps with those from the target interval (filled circles: target vs. first comparison interval; open circles: target vs. second comparison interval). We find a significant negative correlation between the fixation location overlap and thresholds in the fixed-surfaces conditions: Spearman's rank-order correlations, r(8) = −0.78, p < 0.05 for target and first comparison intervals; r(8) = −0.82, p < 0.01 for the second comparison interval. In contrast, we found no significant correlation between the thresholds and fixation overlap in the shuffled-surfaces condition.

**Figure B3:**
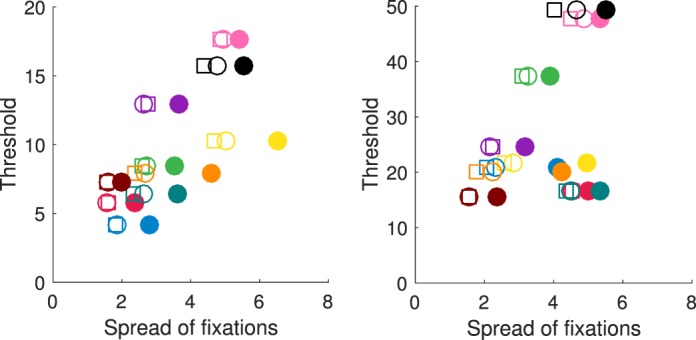
Spread of fixations predicts illumination discrimination thresholds in the fixed-surfaces but not in the shuffled-surfaces condition. Left panel: fixed-surfaces condition. Right panel: shuffled-surfaces condition. For each observer the average illumination discrimination threshold is plotted against the spread of fixations in the target interval (filled circles), first comparison interval (open circles), and second comparison interval (open squares). We find a significant correlation between the spread of fixations and thresholds in the fixed-surfaces conditions: Spearman's rank-order correlations, r(8) = 0.75, p < 0.05 for target and first comparison interval; r(8) = 0.88, p < 0.01 for the second comparison intervals. In contrast, we found no significant correlation between the fixation spread and fixation in the shuffled-surfaces condition.

**Figure B4:**
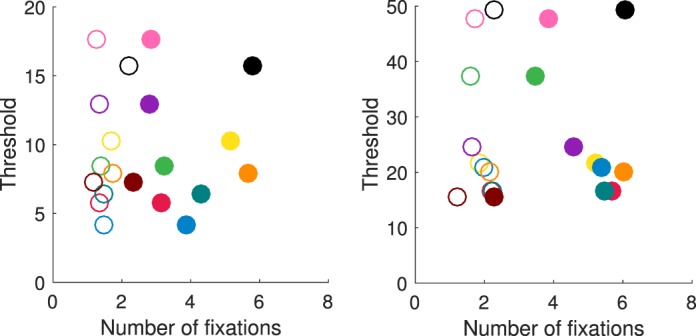
Number of fixations does not predict illumination discrimination thresholds. Left panel: fixed-surfaces condition. Right panel: shuffled-surfaces condition. For each observer the average illumination discrimination threshold is plotted against the number of fixations in the target interval (filled circles) and the comparison intervals (averaged across intervals; open circles). The data show that the number of fixations (in either comparison or target intervals) does not predict thresholds in either the fixed-surfaces or shuffled-surfaces condition.

## Footnotes

1 The image-based illumination *XYZ* value was obtained by rerendering the target scene with two perfect reflectors placed into it (one on the floor, one on the back wall) and averaging the *XYZ* values of the light reflected from these perfect reflectors, after passing the rendered images through the same processing pipeline (from hyperspectral to LMS to scaled RGB) that we used for the experimental stimuli.

## Appendix A: Experiment 1A

Here we present the results of Experiment 1A, where in the shuffled-surfaces condition one surface remained fixed across stimulus scenes while all the remaining surfaces varied. The omission of one surface from across-scene shuffling occurred due to a bug in the experimental code (see the note in Experiment 1, Results section).

### Methods

The methods were identical to those in Experiment 1, with two exceptions. First, one of the surfaces in the scene remained fixed and did not vary across stimulus scenes in the shuffled-surfaces condition (see Figure A1). This surface had a pinkish-appearing (physically realistic) reflectance, which was assigned by default in the rendering pipeline. This reflectance was not one of the preselected 14 surface reflectances that we intended to assign to the tiles in the scene (we provide this reflectance in the surface reflectance table in the online supplement under the column header “BlenderReflectance”).

Second, the control computer was upgraded with a faster (SSD) disk drive for Experiment 1. This may have had a minor effect on Experiment 1A stimulus timing parameters relative to those reported for Experiment 1.

In Experiment 1A, the mean luminance of the target scene was 16.50 cd/m^2^ in the fixed- and 16.39 cd/m^2^ in the shuffled-surfaces condition (*SD*s: 0.002 and 0.5, respectively). The mean image chromaticity was *xy* = (0.326, 0.316) in the fixed- and *xy* = (0.324, 0.320) in the shuffled-surfaces condition, *SD*s: (<0.0001, <0.0001) and (0.0068, 0.0039). The mean pairwise ΔE difference across all versions of the target image in the shuffled-surfaces condition was 3.55 (*SD*: 1.78; maximum: 8.42).

A document that describes the experimental design and the data analysis plan for this study was registered before the start of data collection and is available at https://osf.io/csuza/.

#### Observers

Eleven observers participated in Experiment 1A (eight females and three males; age: 18–21). They all had normal or corrected-to-normal visual acuity and normal color and stereo vision, as assessed via standard lab procedures (described above). One observer (male, age 20) was excluded from the experiment after his third session because his thresholds in the fixed-surfaces condition fell outside of the stimulus range (for both red and yellow chromatic directions in the second fixed-surfaces session).

All but one observer completed the same questionnaire as used for Experiments 1 and 2 at the end of the last session (the questionnaire was introduced into the experimental procedures only after the first observer had already completed the study). Scanned questionnaires with observers' responses are available in the online supplement.

### Results

Observers' thresholds in the fixed- and shuffled-surfaces conditions of Experiment 1A are shown in Figure A2 (squares). Results of Experiment 1 are shown for comparison in panel A (circles). Overall, the data from the two experiments are very similar. To investigate this more quantitatively, we compared the results of Experiments 1 and 1A using a three-way ANOVA with experiment as a between-subject factor and condition and illumination-change direction as within-subject factors. The results of the two experiments were essentially identical: We did not find a significant main effect of experiment, *F*(1, 17) = 0, *p* = 0.97, or any significant interaction involving Experiment: Experiment × Condition interaction, *F*(1, 17) = 0.36, *p* = 0.56; Experiment × Illumination Direction interaction, *F*(3, 51) = 0.3, *p* = 0.83; Experiment × Condition × Illumination Direction interaction, *F*(3, 51) = 2.04, *p* = 0.12. Based on this we conclude that the lack of experimental control over a single surface in Experiment 1A had no measurable effect on thresholds. This conclusion is the basis of our decision not to rerun Experiment 2.

For completeness, we present a more detailed analysis of the results of Experiment 1A. This analysis confirms the main conclusions we drew from Experiment 1. First, thresholds in the shuffled-surfaces condition are elevated with respect to those in the fixed-surfaces condition. Second, thresholds for the blue illumination-change direction are overall the highest. There are some between-experiment differences in significance of Condition × Illumination interaction and pairwise comparisons of thresholds between illumination-change directions. We have recorded this kind of between-experiment difference previously (Radonjić et al., 2016), and we consider them to reflect natural experiment-to-experiment fluctuations in small and inconsistent effects.

#### Detailed results of Experiment 1A

In Experiment 1A all 10 observers were able to complete the illumination-discrimination task in the shuffled-surfaces condition (the thresholds in each illumination-change direction were within stimulus range in at least one session). For three observers thresholds were out of range in one session: Three different directions for one (blue, green, and yellow), two different directions for the two remaining observers (blue and yellow; blue and green). Similar qualitative results were obtained in Experiment 1: Thresholds for nine out of 10 observers fell within a stimulus range in at least one session and for three out of nine remaining observers thresholds were out of range in one session: all four directions for one observer, three (blue, green, and red) directions for another observer, and one (blue) direction for the remaining observer.

We quantified the effect of surface shuffling on observers' thresholds in Experiment 1A using a two-way repeated measures analysis of variance (ANOVA) with condition and illumination-change direction as fixed within-subject factors. As in Experiments 1 and 2, shuffling the reflectance of surfaces in the scene had a large effect on illumination discrimination thresholds: main effect of condition, *F*(1, 9) = 27.14, *p* = 0.001. Overall thresholds increased by 19.6 ΔE units (28.6 ΔE in the shuffled- vs. 9 ΔE units in the fixed-surfaces condition). The increase in thresholds from the fixed- to shuffled-surfaces condition held for all observers and illumination-change directions, as illustrated in Panel B of Figure A2.

As in Experiments 1 and 2, the degree to which surface shuffling affected performance varied across observers (from 6.3 to 35.2 ΔE). A posthoc ANOVA equivalent to the one described above but with the addition of Observer modeled as a random factor (not included in the preregistration plan for this experiment) revealed a significant Observer × Condition interaction, *F*(9, 27) = 30.97, *p* < 0.001 (neither the main effect of observer nor any other Observer interactions were significant).

Thresholds differed significantly across different illumination-change directions: main effect of direction, *F*(3, 27) = 22.26, *p* < 0.001, but unlike in Experiment 1 and 2, the pattern of threshold variation across directions differed in the two conditions: Condition × Illumination Direction interaction, *F*(1.9, 17.2) = 6.81, *p* < 0.01. To gain better understanding of this interaction, we analyzed the change of thresholds across directions separately for each condition (a separate one-way repeated measure ANOVA for each condition). In both conditions, (a) thresholds varied significantly across directions, main effect: *F*(3, 27) = 9.69, *p* < 0.001 in the fixed-surface and *F*(2.1, 18.73) = 17.41, *p* < 0.001 in the shuffled-surfaces condition; and (b) sensitivity to changes in the blue chromatic direction was worst (thresholds were the highest). In the shuffled-surfaces condition thresholds in the blue direction were significantly higher than those in the red (*p* < 0.005), yellow (*p* < 0.001), or green direction (*p* < 0.001; as shown via Bonferroni-corrected posthoc tests, with significance level adjusted for multiple comparisons to *p* = 0.05/6 = 0.0083). In the fixed-surfaces condition, thresholds were also significantly higher in the blue than in the red direction (*p* < 0.001; blue-green and blue-yellow difference did not reach significance). In addition, for the fixed-surfaces condition, thresholds were higher in the green than in the red direction (*p* < 0.007). In the shuffled-surfaces condition this difference was in the opposite direction but did not reach significance.

## Appendix B. Additional analyses of eye-fixations (Experiment 2)

We analyzed the degree to which observers tend to fixate at the same locations in the image across intervals of the illumination discrimination task. This analysis can be considered a location-driven, rather than reflectance-identity-driven version of the analysis shown in Figure 8.

As in the reflectance-based analysis, we computed the proportion of fixations in each comparison interval that overlapped with a fixation from the target interval, where we considered two fixations to overlap if they were within 1° of visual angle of each other. For each observer and condition, the left panel in Figure B1 shows this proportion in the shuffled-surfaces versus the fixed-surfaces condition (filled symbols indicate first comparison interval; open symbols indicate second comparison interval). This number is generally lower in the shuffled-surfaces condition, indicating that where observers look across trial intervals varied more in the shuffled-surfaces condition, although for most observers the effect is small: paired *t* tests: *t*(9) = 2.35; *p* < 0.05 for the target versus first and *t*(9) = 2.42; *p* < 0.05 for the target versus second comparison interval.

To examine whether the number of trials where the observers looked at the same location across intervals (by our measure) was greater than one would expect by chance, we also recomputed the same statistic but with fixation data for each comparison interval shuffled across trials. A different shuffling was used for each of the two comparison intervals. The results are shown in the right panel of Figure B1. For all observers, trial-shuffling reduces the proportion of fixation overlap considerably but not to zero, suggesting that observers tend to look at the same general part of the stimulus scene across trials.

Parallel to the analysis shown in Figure 9, the degree of fixation location overlap within a trial predicts thresholds in the fixed-surfaces condition but not in the shuffled-surfaces condition (Figure B2). Further, the overall spread of fixations is correlated with the thresholds in the fixed-surfaces condition, but not in the shuffled-surfaces condition (Figure B3). Finally, Figure B4 shows that there is no significant correlation between the overall number of fixations and thresholds in either the fixed-surfaces or the shuffled-surfaces condition.

## Appendix C: Deviations from preregistered plan for data collection and analysis

Bellow we summarize the deviations from the preregistered plans for data collection and analysis for each experiment. Preregistrations for later experiments incorporated information obtained from earlier experiments. For each preregistration deviation listed below we note in the parentheses the experiment(s) it refers to.

Changes in experimental procedures:

- The preregistration document stated that we will only enroll observers who are “older than 18.”This was a typo; we intended to write “18 or older.” Three observers in Experiment 1A were 18 (Experiment 1A; Experiment 2).
- Observers completed a postexperimental questionnaire at the end of the last session. The questionnaire was introduced in the course of Experiment 1A but only after the first observer had already finished the experiment (Experiment 1A).
- We excluded and repeated one fixed-surfaces session for one observer due to technical difficulties in displaying the stimuli. We suspect that the stimulus which was intended to be viewed stereoscopically was displayed only on one screen while the other screen was black (Experiment 1).

Changes in data analysis:

- We did not initially specify how we would average thresholds across sessions in the shuffled-surfaces condition when the threshold for a given illumination-change direction fell outside of the stimulus range in only one session. The procedure we used was established after data collection in Experiments 1A and 2 (Experiments 1A and 2).
- We conducted a qualitative comparison of out of range matches between Experiment 1 and Experiment 1A (Experiment 1).
- We estimated the performance of a randomly responding observer to compare it to the performance of our observers in the shuffled-surfaces condition (All experiments).
- The preregistration document stated that thresholds larger than 50 ΔE require exclusion as they fall outside of the stimulus range. It was not, however, specified that this number should refer to the nominal 50 ΔE value. The exact value (in ΔE) that corresponds to the nominal 50 ΔE units varies across illumination-change directions (Experiments 1A and 2).
- To investigate whether observers differ in performance in our experiment (either the fixed- or shuffled-surfaces condition), we ran a posthoc ANOVA (with condition and illumination-direction as a fixed within-subject factors), which also included Observer as a random factor (Experiments 1A and 2).
- In some instances, our preregistered data analysis plan did not describe our intended procedures in sufficient detail. Specifically, to investigate the significant Condition × Illumination Direction interaction in Experiment 1A, we conducted two separate one-way ANOVAs (one for each illumination change direction). Further, as a follow-up analysis to ANOVA which showed a significant main effect of illumination-change direction, we conducted pairwise comparisons of thresholds across different illumination change directions in Experiment 1A (on one-way ANOVAs only) and Experiment 2 (Experiments 1A and 2).
- As noted in the data analysis section of Experiment 2, we used the Nyström/Holmqvist fixation algorithm, to identify fixations from the recorded observers' eye positions, instead of the built-in Eye Link algorithm. The Nyström/Holmqvist algorithm was more sensitive to small changes in observers' fixation typical of our study, particularly during the comparison intervals, which were fairly short (Experiment 2).
- The preregistration did not specify any trial exclusion criteria based on the loss of eye position data (Experiment 2).
- The analysis of the number of fixations is conducted roughly as described in the preregistration, except that it included interval (target vs. comparison) as a fixed within-subject factor, in addition to factors condition (fixed, within-subject) and observer (random; Experiment 2)
- The analyses of fixation spread and location are exploratory, as is the surface-based analysis of fixations. The preregistration document indicated that we would conduct exploratory analyses of fixation data, but did not describe in detail the specific form that these analyses would take (Experiment 2).
- The analyses correlating observers' performance in the fixed- versus shuffled-surfaces condition with the eye fixation measures are exploratory and were not described in the preregistration. The same is true for the analysis examining the correlation of observers' performance in the fixed- and shuffled-surfaces condition.

Other changes:

- We plan to describe modeling work, which focuses on the information for illumination discrimination available in the stimulus and the signals in the early visual pathways, in a separate manuscript (Experiment 2).
